# Electromagnetic Field Stimulation Effects on Intrinsically Disordered Proteins and Their Role in Aging and Neurodegeneration

**DOI:** 10.4236/jbise.2025.1810030

**Published:** 2025-10-29

**Authors:** Felipe P. Perez, Joseph Bandeira, Jorge Morisaki, Haitham Kanakri, Maher Rizkalla

**Affiliations:** 1 Department of Medicine, Division of General Internal Medicine and Geriatrics, Indiana University School of Medicine, Indianapolis, IN, USA;; 2 Department of Bioengineering, REMFS LLC, Indianapolis, IN, USA;; 3 Department of Electrical and Computer Engineering, Purdue University, Indianapolis, IN, USA

**Keywords:** Alzheimer’s Disease, Treatment, Electromagnetic Fields Stimulation, Intrinsically Disordered Proteins, Quantum Mechanics, Posttranslational Modifications

## Abstract

There is increasing evidence from preclinical studies. There is growing evidence from preclinical studies in cell cultures and small organisms that exposure to Electromagnetic Fields (EMFs) produces beneficial biological effects. However, controversy persists due to the absence of a clearly defined mechanism. Classical physics, constrained by the non-ionizing nature of these exposures, cannot account for these effects, which do not involve the breaking of chemical bonds to induce conformational changes in proteins. Emerging studies suggest that these effects are mediated through quantum mechanical phenomena—specifically, quantum tunneling and particle-wave duality—acting on the water surrounding proteins at their interfaces. Furthermore, we present evidence of EMF-induced conformational changes in Intrinsically Disordered Proteins (IDPs), including beta-amyloid, tau, alpha-synuclein, and Heat Shock Factor 1 (HSF1). These findings offer a new framework for understanding EMF bioeffects and open promising avenues for research in biophysics and quantum biology. In this context, we address the challenge of reproducibility by examining how variables such as frequency, intensity, Specific Absorption Rate (SAR), and exposure time windows interact, along with how parameters like polarization, phase, pulse modulation, and scheduling influence outcomes. Experimental data identify specific RF frequencies and SAR levels that activate proteostasis and autophagy in cell cultures and small animal models, with potential applications in human treatments that remain consistent with safety thresholds established by regulatory agencies.

## 1. INTRODUCTION

Quantum EMF radiation involves photon particles as the fundamental carriers of discrete energy. The radiation can be described using quantum mechanics rather than Maxwell’s equations because the field’s energy is quantized into distinct photon units. Although photons are massless, they act as carriers of EM forces that interact with human tissues. Studying EM quantum and quantum biology may help us understand how energy transfers via photons in water-rich human tissues [1]. Quantum technology is already used in MRI machines, which interact more effectively with soft tissues to produce clearer images. This technology can potentially be expanded to address brain cell disorders, such as Alzheimer’s disease, serving as a future application. Research on quantum EM in the human brain could lead to new approaches for treating Alzheimer’s [2]. It might also provide new insights into the role of amyloid fibrils, which are linked to the cause of Alzheimer’s disease [3].

The primary focus of the EMF quantum effects is on interfacial water, which was traditionally seen as a passive background for biochemical reactions. It is now increasingly understood as an active participant in biological processes, especially at the quantum level [4]. By highlighting this parallel, we postulate that interfacial water domains play a catalytic role in cellular energetics, mediating proton transfer comparable to enzyme active sites [5]. The difference is that water’s “active site” is not a single pocket in a protein but a dynamic quasi-two-dimensional sheet enveloping membranes, proteins, and nucleic acids. Within this sheet, protons might migrate through tunneling from one site to another. Proteins are not merely static structures; they are deeply entwined with their hydration shells, and this relationship is especially pronounced in Intrinsically Disordered Proteins (IDPs) [6]. Unlike folded proteins, IDPs lack a stable three-dimensional conformation and instead exist as dynamic ensembles of conformers. This structural plasticity is fundamental to their biological roles as molecular hubs in signaling networks, stress response, and proteome surveillance. Crucially, these roles are modulated not only by molecular interactions but also by the physical and quantum properties of the surrounding water. Structured water, particularly the interfacial form, exhibits semi-crystalline ordering [7], altered charge distribution, and capacity for electromagnetic coherence [8]. Recent advances in quantum biology suggest that interfacial water supports proton tunneling, energy resonance, and field-sensitive organization—all properties that profoundly impact protein-water interactions.

This paper examines the dynamic hydration of IDPs, considering recent developments in quantum biophysics. By integrating spectroscopy, molecular dynamics, and quantum electrodynamics findings, we propose a model in which hydration shells function as quantum-responsive buffers that regulate protein conformation, proteostasis, signaling, and longevity. We argue that oxidative stress during aging damages protein surfaces, disrupting hydrophilic zones and displacing ordered water, causing the breakdown of these hydration structures, leading to Post-Translational Modifications (PTM) during stress processes, affecting transcriptional factors with the result of proteostasis collapse, and protein deposition disease such as AD. In this study, we also discuss potential therapeutic strategies such as repeated electromagnetic field stimulation (REMFS) to repair and reactivate proteins involved in proteostasis and autophagy for the treatment of age-related protein deposition diseases, including Alzheimer’s, frontotemporal disease, and Lewy body disease.

## 2. INTRINSICALLY DISORDERED PROTEINS

IDPs are abundant in all cells and increase with the increase in the complexity of the organism [9]. These proteins are found more frequently in signaling proteins and proteins controlling gene activity, such as transcription factors [10]. IDPs, because of their amino acid sequences with high net charge and low hydrophobicity, do not follow conformational rules for ordered proteins and domains that lose structure and biological function at various environmental conditions, such as temperatures, pH, or EMFs; they gain ordered structure because of the enhancement of the hydrophobic interaction [11]. IDPs have a low content of bulky amino acids and a high proportion of polar and charged amino acids, which promotes hydrogen bonding between the polar atoms of charged residues and water molecules, improving interactions with interfacial water [12]. A lower amino acid alphabet characterizes IDPs compared to the alphabet utilized in the amino acid sequences of ordered domains and proteins [13], and a higher sequence space than that of ordered proteins, which increases the vast structural complexity of these proteins and regions [13]. Also, because of their inability to fold into unique structures in isolation and their globally reduced structural content, parts of an IDP are disordered or ordered at different times and stimuli [14].

Therefore, IDPs are a complex mixture of foldable, partially foldable, potentially foldable, or non-foldable segments [15]. IDPs can have four different conformations depending on the environmental conditions, including folded or ordered, molten globule, pre-molten globule, and coil-like [16]. IDPs’ conformational plasticity predisposes them to their extreme sensitivity to changes in the environment and interaction with a multitude of proteins, and consequently to fold and bind in many different ways [17], which gives them unique properties such as control, regulation, interaction with, as well as being controlled and regulated by multiple types of proteins and RNAs [18]. A multi-funnel structure characterizes IDPs’ free energy landscape, meaning it has various competing low-energy conformations rather than a single, stable, folded structure [19], which reflects the existence of numerous conformations that constitute its dynamic conformation, possessing a very disordered nature [20].

The shape of IDP energy is a multi-funnel-like landscape and can be easily altered by minimal variations in environmental factors, in contrast to the funnel-like energy landscape of an ordered protein, where the hilly plateau energy landscape of the IDP determines its conformational plasticity and its ability to fold differently depending on environmental factors. For this reason, IDPs can easily participate in cell regulation, signaling, and control, thereby serving as crucial regulators of various cellular processes [21]. All these characteristics predispose IDPs to develop Post-Translational Modifications (PTMs), which are chemical changes affecting proteins after molecular synthesis. PTMs can be covalently added to amino acids. IDPs use multiple mechanisms to modulate cell function, structural properties, and location of these proteins, including controlled degradation and posttranslational modifications [22].

PTMs induce many changes to IDPs’ structure. PTMs can covalently add chemical groups, carbohydrates, lipids, proteins, or nucleic acids to amino acid side chains of the IDPs [23]. IDPs can also undergo enzymatic cleavage of peptide bonds or removal of various chemical groups; some of these cleavage reactions produce toxic fragments, including aggregation-prone species, while others result in modified proteins with novel functions [24] and can give different functions to the same protein [25]. PTMs are readily reversible because of modifying and demodifying enzymes, and these modifications include phosphorylation [26], acetylation [27], glycosylation [28], lipidation [29], methylation [30], nitration [31], and protonation [32], all of which give conformational variability of IDPs to control protein function [32]. A conspicuous member of IDPs is the peptide amyloid-*β* (A*β*) that aggregates into metal-enriched amyloid-forming plaques in Alzheimer’s disease [33]. Furthermore, genetics, oxidative and nitrative stress, as well as mitochondrial impairment, impact the structural flexibility of the unstructured IDPs to produce disease mechanisms [34]. In addition, simulations indicate that extended conformations are more likely to appear when hydrogen bonds are not stable, rather than when they are stable [35]. For example, IDPs like HSF1 and alpha-synuclein gain a more ordered structure with temperature rise and are more disordered at low temperatures [36]; they also fold in highly acidic or alkaline environments, folding into a more ordered conformation. This structural instability of IDPs/IDPRs is further supported by their well-known exceptional sensitivity to proteolytic degradation [37]. The poor regulation of IDPs is associated with various pathologies, including aging, cancer, neurodegeneration, cardiac disease, and diabetes [38].

## 3. THEORETICAL AND EXPERIMENTAL QUANTUM MECHANISMS IN WATER

Cells are formed of 70% water, which is mostly bulk-like; however, a significant portion of intracellular water is interfacial, specifically a few nanometers of proteins, membranes, RNA, and DNA surfaces [39]. Structured water at biological interfaces (also known as Exclusion Zone (EZ) water) has demonstrated unusual behaviors such as long-range exclusion of solutes and charge separation [40]. Gerald Pollack and colleagues showed that hydrophilic surfaces can induce a several-hundred-micron-thick EZ region in water that is negatively charged and relatively ordered, expelling microspheres and solutes [41]. Notably, EZ water formation is accompanied by the release of protons into the surrounding bulk water, effectively creating a charge separation akin to a battery [40]. The behavior of this interfacial water cannot be explained by classical physics alone; for instance, infrared light dramatically expands the EZ, suggesting that electromagnetic fields directly interact with water’s structuring [42]. These observations have catalyzed new theoretical approaches, such as Quantum Electrodynamics (QED) models, that posit that water can form large-scale coherent domains with electromagnetic properties [43].

Experimental data obtained through ATR-IR spectroscopy, optical microscopy, and electrokinetic measurements confirm that water adjacent to hydrophilic interfaces organizes into highly ordered regions known as Exclusion Zones (EZs) or coherence domains. These zones exhibit semi-crystalline molecular alignment, a strong negative charge, and proton exclusion into the adjacent bulk water, creating a substantial electric potential of up to −200 mV across the domain [44]. This charge separation establishes an internal electric field that persists over micrometer distances, behaving similarly to a microscopic capacitor. The stability of these structures requires a continual energy input, primarily from infrared radiation, which has been shown experimentally to expand the size and stability of EZs [45]. The underlying electrostatic potential field can be modeled by the classical Poisson equation:

$$\nabla^{2}\varphi =-\rho /\varepsilon$$

where *φ* is the electrostatic potential, *ρ* is the local charge density, and ε is the permittivity of the medium. This classical field description accurately captures the observed potential distribution within and around the EZ. Further experimental evidence supports that low-frequency electromagnetic fields (e.g., 50 – 75 Hz) can alter EZ structure in a non-thermal manner, consistent with resonant coupling to the hydrogen bond network [46]. Beyond classical descriptions, Del Giudice and Preparata proposed that coherence domains in water reflect a mesoscopic Quantum Electrodynamics (QED) phase, wherein water molecules oscillate in phase with a self-trapped electromagnetic field [47]. Although this theoretical model elegantly explains certain long-range ordering phenomena, it remains a theoretical extension beyond the directly observed experimental evidence. Thus, for biological integration, the structured water experimentally validated electrostatic properties provide a sufficient foundation. However, experimental data obtained by ATR-IR spectroscopy are fully compatible with Quantum Electrodynamics (QED) predictions that induce a coherent state in the water [48]. It has shown that electromagnetic fields facilitate the interactions among non-polar molecules, and a density threshold with a critical temperature, also induce the appearance of a coherent state of the matter in that particles oscillate coherently between two quantum states giving rise to a long-range attraction among atoms or molecules that adds up to their electrostatic properties with net negative charge, excluding protons into the bulk. This charge separation creates an internal electric field and provides a medium for quantum coherence [49].

Furthermore, another study demonstrated that liquid water must be a two-phase system, that according to QED, part of the molecules are organized in collective coherent vibrational domains [50]. Their energy range corresponds to the spectral distance between the two energy levels at which the electron of each water molecule oscillates. The second phase comprises molecules set out of tune by thermal fluctuations that push components out of the coherent state. In addition, a meta-analysis showed a new order parameter characteristic for water molecule assembly, which implies quantum coherence and entanglement. This indicates water molecule assembly shows electromagnetic and electronic collective states that contain “quantum imprints or molds” because water molecules are ordered in a partially distorted tetrahedral geometry, which yields a specific network structure [51]. This water quantum coherence is stabilized by interaction with biomolecule surfaces [52] and posits that at room temperature, about 40% of cell water is in a coherent phase. Thus, every cell protein, DNA strand, or membrane is likely wrapped in a sheath of coherent water, where the water molecules are aligned and organized in a specific way rather than being randomly arranged like in regular water Within RNA, the initial layer of interfacial water is viewed as integral part of the nucleic acid structure because it influences folding, architecture, and intramolecular interactions [53]. This hydration layer modulates biological activity, as many of water’s notable properties stem from its hydrogen-bonding network [54]. Key cellular properties of this hydration layer include cooperative hydrogen bonds, proton currents, osmotic effects, hydrostatic pressure, density shifts, and selective ion exclusion. Such characteristics result in stronger, shorter hydrogen bonds in interfacial water, which has a greater heat capacity than bulk water since more energy is required to disrupt these bonds [55]. When considering the cell as a network of biomolecules, we must include their water shells; we have a network of articulate water domains pervading the cell and connecting at interface boundaries. The arrangement of interfacial water is influenced by the nature of electrostatic forces at the biomolecular surface [55]. The initial hydration layer is under unique quantum confinement, as polar and charged groups of amino acids and nucleic acids engage with surrounding hydrogen bonds [56] from the interfacial water. Such charged and polar regions generate electric fields [57]. Such polar and charged groups are sources of electric fields [58], which play a key role in proton transfer [59]. This confined, electrokinetic water demonstrates quantum tunneling behavior [60]. In structured hydration shells, similar tunneling might facilitate rapid proton translocation and charge redistribution [61], which is essential for signaling and energy transfer. Proton tunneling in this lattice mirrors the quantum mechanical effects seen in enzyme catalysis, where tunneling accelerates reaction rates by orders of magnitude [62]. Moreover, H-bonds oscillate collectively when these frequencies align with external EMFs, and coherent resonance is possible, enhancing water’s ability to store and transmit information [63].

This coherent water can profoundly affect biomolecular dynamics, particularly in Intrinsically Disordered Proteins (IDPs) such as amyloid-*β*, *α*-synuclein, tau, and Heat Shock Factor (HSF1). Because IDPs are highly reliant on stable, tightly bound hydration shells to maintain their flexible, functional ensembles, the disruption of the structured hydration layer can expose hydrophobic residues, facilitating pathogenic aggregation. Experimental work shows that around tau amyloid fibers, hydration water becomes significantly more mobile compared to soluble tau, favoring aggregation through an entropy-driven mechanism [64]. Similarly, simulations reveal that the expulsion of structured water upon protein misfolding contributes thermodynamically to amyloid assembly [65]. Thus, coherent, charge-separated water at the protein interface acts as a stabilizing matrix, suppressing aggregation and preserving proteostasis. In the context of aging and disease, loss of hydration coherence might tip this delicate balance, accelerating misfolding and dysfunction in critical IDPs such as amyloid-*β*, tau, *α*-synuclein, and HSF1 [66].

## 4. THEORETICAL AND EXPERIMENTAL EMF AND PROTON TUNNELING EFFECTS

Studying water behavior under EMF is difficult because hydrogen bonds have lifetimes on the order of picoseconds, which are shorter than the time resolution of methods like Nuclear Magnetic Resonance (NMR) or dielectric spectroscopy [67]. In one EMF experiment, delivering an intense Terahertz (THz) pulse with a peak electric field of 14.9 MV/cm to liquid water enhanced hydrogen-bond stretching and bending motions, triggering proton tunneling [68]. Quantum tunneling is a phenomenon where particles pass through energy barriers that they would not be able to pass through classically. This occurs because of the probabilistic nature of quantum mechanics [69]. In the context of water molecules, quantum tunneling can influence the dynamics of hydrogen bonding and molecular rearrangements. Proton tunneling is a quantum mechanism highly relevant here. A proton in a hydrogen bond can tunnel through the energy barrier between two equilibrium positions (for example, between a donor and acceptor oxygen in adjacent water molecules). In bulk water, this underlies the Grotthuss mechanism, giving rise to anomalously high proton mobility (proton conductivity) [70]. In structured water at interfaces, several factors can enhance tunneling probability. First, the strong ordering and shorter hydrogen-bond distances in EZ water (with tightly aligned molecules) lower the proton transfer barriers [71]. External perturbations—such as an oscillating electromagnetic field—can further modulate the hydrogen bond length, potential energy landscape, and quantum tunneling [72].

Additional evidence that polarized EMF radiation exerts biological effects comes from observations that it can dissociate water into its elemental components [73]. In one study, Rao exposed distilled water to a polarized 2.45 GHz field and found, via Raman spectroscopy, marked changes in the O–H stretching vibration. These alterations promoted proton tunneling and protonation of nearby molecules. Notably, even when using very different exposure conditions (THz vs. Hz), other investigators reached similar conclusions that various EMFs can cause water dissociation [74]. EMF exposure can also reinforce and shorten hydrogen bonds at biomolecular surfaces. For instance, when hemoglobin and bovine serum albumin in aqueous solution were subjected to a 50 Hz field, the Amide II absorption band increased and shifted toward higher energies. This was interpreted as strengthening of hydrogen bonds within the proteins’ secondary structure [75]. While EMFs raise the rotational kinetic energy of bulk water, the most pronounced effects occur in the first hydration shell surrounding biomolecules. In this layer, water molecules are fixed in orientation and rotate with difficulty. Under EMF, however, they can execute large-amplitude oscillations [76]. At the molecular scale, EMF oscillations cause water molecules in the first interfacial layer to attempt to realign their dipole moments with the polarity of the incoming radiation. This dipole reorientation perturbs the hydrogen bonds linking the interfacial water to biomolecules [77].

In biological tissues, all polar molecules, including water, can be forced to oscillate in phase with an applied field, with motion constrained to planes parallel to the field’s polarization [78]. This represents a key factor in the quantum effects observed with REMFS: artificial polarization of excitatory oscillations occurring when REMFS interacts with interfacial water in a biological system. These oscillations occur at a lower frequency than that of the quantum system being exposed, shifting the system’s natural frequency toward the excitatory frequency [79], much like a driven harmonic oscillator [80]. In this analogy, the oscillator receives continuous input from an external force [81]. And if the excitatory frequency is slower, the oscillator’s own frequency adjusts downward toward the excitatory frequency [81]. EMF exposure shortens hydrogen-bond lengths and increases bond angles in water as a result of large-amplitude motions [82]. These motions reduce the O−H⋯O hydrogen-bond distance in the first hydration layer surrounding nucleic acids to less than 1.85 Å [83], an ideal distance for increasing the probability of proton tunneling [84]. The magnitude of this effect varies with the molecule’s position relative to the field. Consequently, hydrogen-bond energy and geometry shift as front-line water molecules rotate [85], producing anisotropic changes in the hydrogen-bond network [86] and hydrogen pairs with a net dipole moment. This reorientation plays an important role in quantum-scale processes such as proton transfer [87], proton transport [88], and the hydration of RNA or IDPs required for their proper function [89].

The protonation state of amino acids, particularly those with titratable side chains (like histidine, lysine, cysteine, and aspartic acid), can change with EMF exposures. This alters the protein’s overall charge and can affect its stability and interactions with other molecules, causing posttranslational modifications [90], as charge is a major driving force for many molecular interactions. Protonation can lead to conformational changes in IDPs because of the impact of electrostatic interactions on their dynamics, affecting their function [91]. The dynamic nature of IDPs means that they can exist in multiple tautomeric forms, which are structural isomers that differ by the position of a proton and a double bond [92]. Multiple studies indicate that EMF exposure can drive proton tunneling events that lead to tautomer formation in nucleic acids. Cerón-Carrasco demonstrated, using Quantum-Mechanical (QM) calculations, that electric fields promote proton transfer resulting in tautomer generation [93]. The work further concluded that, in the presence of electric fields, guanine–cytosine base pairs uniquely satisfy the kinetic requirements for viable tautomer formation. In a later simulation study employing a more structurally accurate DNA fragment, Cerón-Carrasco reported that elevated electric field strengths stabilized tautomeric forms relative to canonical bases [94]. Classical molecular-dynamics simulations also revealed that continuous field exposure can alter nucleic acid conformations within 10 picoseconds [95]. Such tautomers [96] play roles in numerous RNA functions [97]. Inter-conversion among tautomeric states can yield diverse secondary structures that underpin biological processes such as RNA folding, DNA replication, chromatin packaging, and transcription [98, 99]. These structural shifts often facilitate binding to activator proteins, thereby modulating transcriptional and translational activity.

## 5. IDPS AND HYDRATION WATER DYNAMICS

Water dynamics around IDPs play key roles in protein function, including enzyme activity and allostery. IDPs are characterized by high conformational entropy and a preponderance of charged and polar residues. IDPs show higher hydration water density as compared to the globular protein, and the water molecules are tetrahedrally ordered (more local structural order) around disordered regions/proteins as compared to ordered regions/globular protein because of higher mean net charge, promoting stronger water-protein interactions in disordered regions/proteins [100]. This composition attracts extensive hydration shells, resulting in bound water layers that exhibit restricted dynamics, due at least in large part to a greater presence of charged groups in disordered regions [101]. A large fraction of IDPs contains positively and negatively charged residues, depending on the content of charged residues. This is reflected in a hilly energy landscape, where the highs are barriers to forbidden conformations [102]. The distribution of charges and their sequence likely determine the response to environmental changes, so disordered parts respond differently to similar stimuli [103], playing vital roles in protein aggregation, participating in protein-protein interactions, and undergoing binding-induced folding [104]. Another crucial factor for the biological activity of proteins is the internal dynamics, which provides some degree of conformational flexibility and structural dynamics required for catalytic activity [104]. These structured hydration layers are not passive—they interact with both endogenous and external electromagnetic fields, modulating coherence within and across protein complexes [105].

These hydration shells stabilize disordered regions and act as mediators of protein-protein interactions, allostery, and phase separation. Reid et al. showed that negatively charged residues within IDRs form long-lived hydrogen bonds with water molecules, reducing water mobility compared to structured protein surfaces [6]. This behavior suggests a strong coupling between electrostatics and hydration dynamics. IDPs’ function depends on their interactions with other proteins; it needs tight coupling of experimental and computational methods because they do not have a single conformation, and their models require ensembles of conformations representing a distribution of states that the protein adopts in solution [106]. Studies using molecular dynamics simulations and dielectric spectroscopy indicate that water molecules around IDPs exhibit more heterogeneous rotational and translational dynamics than water around structured proteins [107]. This means that water molecules around IDPs are, on average, more restricted in their movement than water around structured proteins. This restricted motion is attributed to a more significant fraction of tightly bound water molecules in the first hydration shell around IDPs. This electrokinetic confined or trapped water exhibits a quantum tunneling behavior [108] linked to signaling and regulatory processes [109]. We propose a conceptual model in which Intrinsically Disordered Proteins (IDPs), structured water, and quantum coherence operate as a highly integrated signaling system. IDPs are enveloped by hydration shells composed of ordered, polarized water that exhibit hydrogen bond resonance and facilitate proton tunneling. IDPs are also involved in stress granules, nucleoli, and other condensates that undergo hydration-modulated transitions from fluid to gel to solid states—progressions associated with aging and neurodegeneration [38]. These hydrogels [110] are composed of uniformly polymerized amyloid-like fibers [111].

## 6. AGE-RELATED BREAKDOWN OF HYDRATION COHERENCE IN IDPS

Intrinsically Disordered Proteins (IDPs) are characterized by high conformational variability, giving them flexible hubs involved in the signaling and regulation of many biological processes, including the aging process [112]. A recent protein-protein interaction network analysis showed that IDPs are found in different clusters associated with several aging hallmarks [113], including genomic instability, telomere attrition, epigenetic alterations, loss of proteostasis, and stem cell exhaustion [114]. This is of prime importance because many IDPs are implicated in a wide range of diseases, including Alzheimer’s, Parkinson’s, Huntington’s, ALS, frontotemporal dementia, cancer, diabetes, and cardiovascular disease [113]. IDPs contribute to disease through several mechanisms, including misfolding and aggregation, altered signaling, and disrupted protein interactions [115]. Some examples of IDPs in disease are Amyloid-beta, which is a key protein involved in Alzheimer’s disease, Tau protein, which is also implicated in Alzheimer’s and other neurodegenerative disorders, *α*-Synuclein, a protein associated with Parkinson’s disease and Lewy body dementia, and p53 and BRCA1 associated with cancer [116].

Quantum coherence might play a role in the folding process of IDPs, allowing them to explore multiple conformations simultaneously before settling into a stable structure [117]. While an intuitive picture for classical coherence is a recurring pattern, quantum mechanical coherence is exemplified by superposition states [118], and it is generated by the interaction of the protons and the heteronuclei [119]. A study with 1H-15N Heteronuclear Single Quantum Coherence (HSQC) spectrum of the intrinsically disordered C-terminal domain of a virus nucleoprotein showed low signal dispersion in the 1H dimension, that would normally result in an increased spectral overlap; however, this low signal dispersion is to some extent counter-balanced by the narrowed line widths of the NMR signals of IDPs because of their inherent dynamic nature [120]. As coherent water structures decay, so does the efficiency of proton tunneling, electron transfer, and protein dynamics [121]. IDPs, heavily reliant on hydration-mediated interactions, lose quantum coherence and begin to misfold and aggregate [122]. In the unfolding and refolding of the IDPs, coherence could be a component of the protein’s ability to follow “a pathway” in a directed process, countering perturbing actions by molecules not involved in the process [122].

Quantum coherence drives us to untangle mechanisms and dynamics controlled by order and synchronization at a quantum mechanical level to enhance functions or develop new molecular systems [123]. For example, a-synuclein structures occur at 30% - 42% in the trimeric and tetrameric forms and 58% - 70% for the monomeric species by Bayesian analysis because of rapid interconversions, suggesting unfolding and refolding of monomeric and multimeric species on timescales faster than tens of microseconds. The monomeric structures predominate in the NMR spectrum because of their enhanced flexibility compared to their multimeric counterparts [120]. This is supported by a study using 1H-15N Heteronuclear Single Quantum Coherence (HSQC) spectrum in that the proposed tetrameric species superimposes within the experimental error on the spectrum of monomeric a-synuclein from E. *coli* [124].

Also, oxidative stress during aging damages protein surfaces, disrupting interfacial water and IDPs quantum coherence, diminishing the cell’s ability to sustain structured hydration [125]. Coherence here refers to the degree to which an IDP’s different functions or interactions are interconnected or depend on each other. For example, an IDP might have multiple binding sites or be involved in different signaling pathways that are linked or regulated together [126]. A study found that all the proteins associated with oxidative stress-induced JNK signaling and cell death were IDPs with Molecular Recognition Features (MoRFs), Post-Translational Modification (PTM) sites, and Short Linear Motifs (SLiMs) that were associated with the disordered regions [127]. Furthermore, cytoskeletal breakdown alters intracellular architecture, compromising the geometric stability of hydration domains [128].

An important IDP involved in the aging process and proteostasis is the transcriptional factor HSF1, which is a key regulator of heat shock proteins and contains extensive IDRs that respond to hydration state, temperature, and multiple PTMs, including redox status [129]. Its dysfunction under stress and age, because of PTMs, correlates with proteostasis collapse in neurodegeneration [130]. Other IDPs involved in neurodegeneration are A*β*, Tau, alpha-synuclein, and TDP-43 [131].

## 7. INSIGHTS INTO HSF1 REGULATION VIA POSTTRANSLATIONAL MODIFICATIONS

PTMs enable proteins to rapidly adopt different structural and functional states in response to internal and external stimuli [132] these occur more frequently in IDPs because they provide access to modifying and disorganizing enzymes and enable facile interactions with protein modules [133] that specifically recognize and interact with modified residues [134], that is a consequence of the abundance of disordered regions in proteomes. PTMs in IDPs occur more often than three to four modifications per protein [135]. In addition, RNA-Binding Proteins (RBPs) such as HSF1 are significantly enriched in disorder compared with the human proteome [136]. HSF1, a master regulator of proteostasis, is profoundly influenced by hydration dynamics. Its activation involves trimerization, nuclear translocation, and DNA binding [137]—each modulated by IDRs sensitive to redox shifts, temperature, and water structuring. HSF1 undergoes phase separation upon heat shock, and its activity is regulated by Post-Translational Modifications (PTMs), the molecular details underlying HSF1 phase separation, temperature sensing, and PTM regulation. HSF1 surfaces at hydrophobic regions restricting water molecules, thereby limiting the degrees of freedom and decreasing entropy. When proteins undergo phase separation, some associated water molecules are released from the protein because of the decrease in protein surface area, resulting in positive entropy gain [138]. Recent findings reveal that Heat Shock RNA-1 (HSR1), a long non-coding RNA, facilitates HSF1 activation by promoting this trimerization [139]. Because RNA structure and interaction affinity are hydration-dependent, HSR1 adds a hydration-sensitive layer to HSF1 regulation. PTM sites that modulate HSF1 activity are not primarily located in Leucine Zipper (LZ) regions but are instead concentrated in the disordered Regulatory Domain (RD) [130, 140]. HSF1’s heptad repeats (HR-A/B/C) are IDR-rich and contain disorder-to-order transitions triggered by thermal stress [109]. HR-C unfolds at elevated temperatures, enabling trimerization via HR-A.

These conformational shifts are fine-tuned by Post-Translational Modifications (PTMs) such as acetylation and phosphorylation, which occur predominantly at hydration-exposed sites that are sensitive to redox state and surface exposure, particularly within intrinsically disordered regions [141, 142]. Oxidative stress accelerates PTMs like CK2-mediated phosphorylation and SIRT1 inhibition, destabilizing HSF1 [143]. SIRT1 inhibition destabilizes HSF1 by impairing its DNA binding ability, leading to a reduced heat shock response and potentially impacting cellular stress responses and aging [144]. This dysregulation is linked to age-related diseases. For instance, Huntington’s disease involves hyperacetylation of HSF1 and impaired mitochondrial function [144]. HSF1 normally activates PGC1*α*, a coactivator promoting mitochondrial biogenesis and thermogenesis [145]. As SIRT1 and PGC1*α* levels decline with age, hydration coherence erodes, further weakening HSF1 functionality [146]. Altogether, this positions HSF1 as a hydration-sensitive quantum responder. It integrates redox signals, thermal fluctuations, and water structuring to maintain proteostasis—an ability compromised during aging and neurodegeneration. However, with aging, these hydration-based networks deteriorate, impairing both classical signaling pathways and quantum coherence mechanisms, ultimately contributing to the loss of proteostasis and cellular resilience.

## 8. EMF STUDIES IN CELL CULTURES AND ANIMALS

### 8.1. Studies in Cell Cultures and Animals

#### 8.1.1. Studies in Animal and Human Immortalized Cell Cultures

A literature review [147] investigated the relationship between EMF and AD at the cellular level. It showed that some have a beneficial effect of EMF exposure on AD manifestations at the cellular level. On the contrary, some studies found no relationship with AD. These discrepancies in the results may be due to differences in the frequency, power deposition, exposure period of EMF, differences in the animal model, cell type [148], and Specific Absorption Rate (SAR). Regulatory agencies recommend using SAR measurements for safety and RF biological effects [149]. For example, short-term EMF exposures activated the A*β* clearance pathway, such as the chaperone-mediated autophagy pathway in human neuroblastoma SH-SY5Y cells, with maximum effect between 30 – 60 minutes of EMF exposure [150]. The ubiquitin-proteasome pathway was activated in primary hippocampal rat neurons exposed to 100 mT for 15 minutes [151]. In another study, HT22 mouse hippocampal neuronal cells and SH-SY5Y human neuroblastoma cells were exposed to 1950 MHz [152] with a high-power Specific Absorption Rate (SAR) of 6 W/kg for two hours per day for 3 days, the levels of APP, A*β* precursor protein cleaving enzyme 1, disintegrin metalloproteinase 10, and Presenilin-1 were not significantly different between EMF exposed culture and controls exposures. Remarkably, this researcher previously found that an SAR of 5 W/kg was beneficial for AD pathology in rats, suggesting an upper limit for the beneficial effects on AD pathology that was not higher than an SAR of 5 W/kg [153].

On the other hand, when IMR-32 neuroblastoma cells were exposed to 60 Hz at 50, 100, and 200 μT for four hours [154], there were no changes in the expression of APP695, an isoform of APP. In a study to assess the expression of proteins involved in the AD pathology (*α*3, *α*5, and *α*7 nicotinic acetylcholine receptors) in SHSY5Y human neuroblastoma cells exposed to EMF, there was no change in expression after exposure to extremely-low frequency EMF [155]. In addition, 50 Hz at 3.1 mT for 18 hours induced A*β* 1–42 secretion [156], and another study showed increased production of prostaglandin E2 and decreased phagocytosis of fibrillary A*β* 42 [157]. All these studies demonstrate the potential harmful effects of EMF during prolonged exposure (>2 hours).

#### 8.1.2. Primary Human Cultures

In our previous study, our lab utilized primary culture because it is directly extracted from human tissue and grown in a laboratory, maintaining its natural characteristics. On the other hand, an immortalized cell line is a genetically modified cell population that can divide indefinitely in culture, often derived from a tumor and therefore different from the natural cell function [158, 159]. Previously, we found that Repeated Electromagnetic Field Stimulation (REFMS) at 50 MHz, exposure times of 5, 15, 30, 60, and 120 min, power of 0.5 W, and a SAR of 0.6 W/kg activated the HSF [160] (master regulator of proteostasis [159] and the autophagy proteins ATG5 and ATG12 [150, 161]) in primary human fibroblasts. Given that the age-related attenuation of HSF1 [137, 162] plays a central role in the process of abnormal autophagy [163] that occurs during aging, it is suggested that EMF interventions to push HSF1 toward its activated state are essential for the activation of autophagy and the clearance of abnormal proteins such as A*β* in age-related diseases [164, 165]. This prompted us to examine REMFS effects on the A*β* levels in primary human brain cultures.

To determine an appropriate EMF dose (dosimetry) [166], we examined the positive and negative effects of REMFS treatments on memory and Alzheimer’s disease (AD) pathology across multiple studies. We used the Inverted U-Shaped Dose-Effect Curve (IUSDEC) for this purpose [167]. Initially, we reviewed literature [168–170] from cell culture, [150, 160, 171–176], animal [177–192], and human [193–197] studies before conducting our own human brain culture experiments. We found that RF power deposition resulting in a SAR between 0.25 and 5 W/kg improves AD pathology and memory. Conversely, SAR levels below 0.25 W/kg or above 5 W/kg [152, 154, 157, 198–209] either had no effect or were harmful to AD pathology and cognition, indicating an IUSDEC. Two human studies support this optimal SAR range: one observed impaired cognitive tasks [210] peed at SAR values below 0.2 W/kg and above 5 W/kg, while accuracy improved within this range [211]. Additionally, longer exposures (5 hours) caused demyelination in mouse neurons, where [212] as shorter exposures (<30 minutes) had no effect [148, 150], suggesting a dose- and time-dependent relationship. Following ICNIRP [149] and IEEE [213] safety guidelines (2 W/kg local head exposure), and considering differences in size, tissue properties, exposure duration, and thermal physiology of animals- as well as the fact that neurons can be harmed even when global SAR stays within safety limits [149] we adjusted the SAR limits for our human brain culture experiments to a maximum of 0.9 W/kg and a minimum of 0.4 W/kg. We then exposed primary human mixed brain cultures (PHB) to various EMF frequencies, exposure durations, daily schedules, and SARs [171] to assess the safety and effectiveness of REMFS on human neurons. REMFS treatment reduced A*β*-40 and A*β*-42 levels without signs of toxicity. Starting treatment on day 7 *in vitro* (DIV 7), we measured A*β*-40 over 14 days. Initially, we used a frequency of 64 MHz with a SAR of 0.6 W/kg for one hour daily; this resulted in a 46% reduction in A*β*-40 compared to untreated cultures. Later, we demonstrated that REMFS at 64 MHz with a SAR of 0.4 W/kg over 14 days produced comparable reductions in A*β*-40 and A*β*-42 levels. Increasing exposure duration from 1 to 2 hours yielded a similar decrease in A*β* levels, establishing the maximum effective exposure time for REMFS. We also identified 0.4 W/kg as the minimum SAR needed to lower A*β* levels, suggesting that the SAR range of 0.04 to 0.9 W/kg could be both effective and safe for human studies.

### 8.2. Animal Studies

Numerous studies have investigated the relationship between EMF and AD in animal models; most of these have been listed in recent reviews [168, 169, 214]. These take into account the various molecular biological mechanisms of AD that have been studied in various animal models, including transgenic AD mouse models and C. elegans. These models are explained in detail in the Ribeiro et al. paper [215]. For example, an AD mouse model exposed to REMFS at 918 MHz with a SAR of 0.25 w/kg for two hours a day for 6 months of treatment showed an improvement in A*β* deposition and cognitive function [180]. Also, REMFS at 918 MHz in the AD mouse model produces an improvement in brain mitochondrial function and an increase in soluble A*β*1–40 following a daily two-hour exposure for a month [179]. An EMF study at 50 Hz, 10 mT for 14 days in AD rats showed improved learning and memory performance [189]. A REMFS at 1950 MHz and SAR of 5 W/kg for 2 h/day in 5xFAD mice and controls showed a significant reduction in A*β* plaques, APP, and CTFs in the hippocampus and entorhinal cortex [153]. A long-term REMFS study reduced hyperactivity and anxiety symptoms while improving memory and increasing glucose metabolism [216]. Another REMFS at 918 MHz study in human and primary rat astrocytes decreased A*β* levels, Reactive Oxygen Species (ROS), H_2_O_2_-induced phosphorylation of p38MAPK and ERK1/2, and mitochondrial ROS, while increasing MMP [172]. REMFS at 1950 MHz [217] and a SAR of 5 W/kg repressed microgliosis genes (Csf1r, CD68, and Ccl6), pro-inflammatory cytokine interleukin-1*β*. And microglial function genes, including Trem2, Fcgr1a, Ctss, and Spi1 in 5 × FAD mice, suggesting that REMFS has beneficial effects in AD pathology and cognition in AD models. Several more recent REMFS studies [218–225] and systematic reviews [226–228] in rodents confirmed the beneficial effects in AD.

On the contrary, prolonged exposure to continuous electromagnetic pulse at 100 Hz and a very high electric field (50 kV/m), for 8 months in Sprague Dawley led to cognitive and memory impairment, increased A*β* level, increased expression of A*β* oligomer and APP, and increased expression of Tau, suggesting a continuous high electric field exposure can cause harmful effects and increase AD pathology [200]. Also, another study at 100 Hz and a very high electric field (50 kV/m), for 8 months in Sprague Dawley, showed an increase in A*β*, BACE1, Tau, and APP in the hippocampus, and cognitive impairment [229]. Another study at 50 Hz and a low magnetic field of 100 μT in Sprague Dawley rats [230] for 12 weeks produced no effects on cognitive function and A*β* level changes, suggesting that a low magnetic field does not have biological effects. Another 15-minute REMFS exposure at 900 MHz and a higher SAR of 6 W/kg exposure showed that RF-EMF did not affect cognition in AD or control rats [231]. REMFS at 1950 MHz and SAR 5 W/kg 2 h/d for 3 months did not improve cognition in AD mice [232], suggesting that a treatment course of 3 months does not affect AD pathology, but longer courses improve cognition and AD pathology [186, 217].

### 8.3. Specific Absorption Dosimetry for Humans

A major limitation in previous studies on the biological effects of RF exposures is the consistency and inconsistencies between the results of the animal and human studies. This is explained by the difference in frequencies, penetration depth, tissue’s dielectric properties, mass density, and complex 3-D E-field distribution, all of which affect the energy absorbed or power deposition on the tissues. SAR measures the energy absorbed or power deposition in the tissues relevant to specific biological effects. Therefore, it is a central consideration for EMF therapy simulations. We cannot determine the SAR only via input power-based estimation due to its dependence on these factors. The conductivity and permittivity of the tissues are important factors; they determine the amount of energy absorbed by the tissue. For example, REMFS uses 64 MHz with a power deposition of a SAR of 0.4 – 0.9 W/kg, which has an appropriate penetration depth for a human head and a SAR consistent with the safety levels recommended by regulatory agencies.

To establish an appropriate SAR framework for human applications, we employed the Inverted U-Shaped Dose-Effect Curve (IUSDEC) to review the literature. Initially, we reviewed the studies from cell cultures, animal and human [233] studies before we performed our human brain culture studies. We found that an RF power deposition with a SAR between 0.25 – 5 W/g improves AD pathology and memory; on the contrary when the SAR was lower than 0.25 W/kg it had no effects, or when it was higher than 5 W/kg, it was detrimental to AD pathology and cognition, suggesting an IUSDEC [233]. Similarly, two human studies support an optimal SAR range; one study found impaired speed in cognitive tasks at an RF field radiation with a SAR of 0.2 and 5 W/kg, in contrast to a SAR of 1 W/kg where accuracy increased. In addition, much longer exposures (5 h) caused demyelination in mouse neurons, and shorter exposures (<30 min) did not have any effects, suggesting a time and dose-dependent effect. Then following the recommendations from the ICNIRP and IEEE Standard for Safety Levels (2 W/kg local head), the differences in size, geometry, long exposure times, and the fact that neurons can be damaged even if the global SAR is within the safety limits, we adjusted the SAR upper limit to 0.9 W/kg for our human brain cultures experiments. In addition, our brain culture studies determined that the minimal SAR required to lower A*β* levels was 0.4 W/kg 41. These suggest that an RF power deposition with a SAR of 0.4 – 0.9 W/kg is an effective and safe framework for human studies [233].

## 9. IMPLICATIONS FOR QUANTUM BIOLOGY AND THERAPEUTICS

Here, we present a hypothesis that reveals a novel domain of quantum biology: the control of the IDPs’ conformation under electromagnetic fields. Electromagnetic fields impact IDPs by influencing their quantum coherence and dynamics, causing water quantum coherence [234] and hormesis, potentially delaying aging and age-related diseases [235]. EMF exposure can cause protonation [33], acetylation [236], phosphorylation [237], decrease reactive oxygen species [238], and potentially reverse PTMs in IDPs. In support of this, EMF exposure induces HSF1 activation in senescent human and rodent cells [160] and delays and reverses cellular senescence [160]. This indicates EMF exposures reverse the HSF1 PTMs that cause the age-related attenuation of the heat shock response and the proteostasis [239], which prevents binding to DNA in senescent cells. EMF exposures also lower A*β* protein aggregation in old rodents [177], supporting that EMF is also effective in older organisms. Furthermore, in a recent EMF study, AML12 and HEK293 cells were exposed to 10 μT for three hours, these exposures increased of HSP70 and HSP90, that was followed by an enhanced of HSP70 and HSP90 for HSP70/HSP90-organizing protein (HOP/STIP1) [236], suggesting that EMF enhances proteostasis because the acetylation of these HSPs reduced protein aggregates and increased cell viability and protein folding [240].

Various studies have examined the effects of EMFs on ROS, which are an important cause of PTMs in IDPs that affect aging and age-related diseases. A study found that EMF exposure at 7 MHz decreased ROS concentrations. They proposed that the ROS production in metabolic processes occurs through singlet-triplet modulation of semiquinone flavin (FADH•) enzymes and superoxide spin-correlated radical pairs. Spin-radical pair products are modulated by the 7 MHz RF magnetic fields that presumably decouple flavin hyperfine interactions during spin coherence [241]. A study found that oscillating magnetic fields alter relative yields of cellular superoxide and hydrogen peroxide, indicating coherent singlet-triplet mixing at the point of ROS formation [125]. This model reframes water not as a background solvent, but as an active participant in cellular information processing. It is noteworthy that the induction of ROS and signs of oxidative stress appear to be more common in neurons exposed to higher SAR and more to ELF-MF than to RF-EMF [242–246]. On the other hand, EMFs and pathological IDPs like A*β* and Tau have a complex relationship, especially in the context of Alzheimer’s Disease (AD) [247]. Several studies have shown that EMF exposure affects A*β* conformation, disrupts existing A*β* fibrils and aggregates, and prevents neuronal dysfunction. EMF at 0.25 V nm-1 causes a conformational shift of A*β* from a pathological *β* to a non-pathological non-*β* shape [248], implying that A*β* can become non-toxic with specific therapeutic strategies. Another EMF exposure at 900 MHz for 3 hours led to a significant reduction in A*β* fibrils in vitro, confirming the simulation results and supporting the idea that EMFs influence A*β* conformation changes [249]. The use of static electric field strengths ranging from 0 V/nm to 0.5 V/nm on an A*β*1–42 supramolecular assembly consisting of a pentamer and a tetramer [250] suggests that these fields induce a permanent or temporary dipole. This dipole causes a conformational change in the protein structure due to the mechanical stress exerted on the charged peptides.

A study investigated the stability of the minimal toxic species, *i.e*., *β*-amyloid dimers, in the presence of an oscillating electric field [251]. The simulation results provide evidence that an external electric field (oscillating at 1 GHz) can disrupt amyloid oligomers, which suggests a possible therapeutic strategy for AD [252]. An EMF study at 70 mV/nm^−1^ GHz found that the diffusion of A*β* oligomers in the membrane decreased, and their interaction with the membrane was also reduced during exposure. Furthermore, at the cellular level, Repeated Electromagnetic Field Stimulation (REMFS) lowers A*β* levels and prevents the formation of oligomers in primary human brain cultures [171]. The proposed mechanism for these effects appears to be proton tunneling caused by the forced vibration from these exposures [148]. A recent study found that magnetoelectric dissociation of Alzheimer’s *β*-amyloid aggregates occurs in ex vivo brain tissues of an AD mouse model [253]. At the organismal level, EMF inhibits A*β* amyloid fibril formation both in vitro and in vivo, alleviating cognitive impairment in Alzheimer’s disease mice [223]. A recent study using 40  Hz gamma frequency showed a significant decrease in A*β* secretion, phosphorylation of AKT, mTOR, and Tau, and A*β* aggregation in SH-SY5Y cells [254]. In an interesting study of Tau protein, a neuroblastoma neuronal cell line was exposed to 4 h of zinc intoxication, which causes Tau-microtubule dissociation. Cel cultures were previously treated with ELF-EMF at 40 Hz and 1 G (with multiple exposure schedules), the showed enhanced microtubule dynamics and increased Tau-microtubule interaction in the face of zinc toxicity, it showed Tau accentuated phosphorylation and reduction in beta tubulin isotypes, depending on electromagnetic frequencies, most pronounced at 3.9 Hz, suggesting that ELF-EMF modulation on the microtubule cytoskeleton essential for microtubule repair [255].

On the contrary, some molecular dynamics simulations suggest that EMF exposures can shift A*β* from a helical to a beta-sheet conformation under nonionizing radiation of varying EMFs [256]. It was shown that the EMFs induce peptide conformations dependent on the field frequency and strength, suggesting that specific ranges of EM field parameters produce well-ordered peptide conformations and modulate the formation of amyloid fibrils. An externally applied Electric Field (EF) of varying strengths (0.1 – 1 V/nm) using molecular dynamics (MD) caused A*β* aggregation, showing that the EF favors the switch of A*β*-peptides from helical to beta-sheet conformation [257]. These results suggest that EMF parameters can be selected as a rational design strategy for AD treatment. All the above suggest that targeting hydration dynamics—via EM field [248], we can modulate IDPs such as HSF1, A*β*, Tau, and other proteins to restore coherence to a non-pathological conformation, consequently delaying proteostasis collapse.

Another dimension worth exploring is the potential impact of electromagnetic harmonics, integer multiples of a fundamental EM frequency, on IDPs. Harmonic components naturally arise in non-linear biological environments and resonate with specific vibrational or electronic modes within IDPs, potentially enhancing or disrupting folding pathways through quantum resonance or interference effects [258, 259]. Such interactions may alter tunneling probabilities or coherence lifetimes, thereby influencing the conformational landscape of these proteins. Understanding the biological sensitivity to not only primary EM frequencies but also their harmonic content could lead to more precise therapeutic targeting and tuning of REMFS protocols [260]. In addition, an important factor is the type of EMF polarization [79], which can be linear or circular. Circularly polarized electromagnetic fields rotate their electric field vector as they propagate, either right-handed or left-handed (chirality). This EMF chirality interacts especially with chiral molecules like proteins and DNA [261–263], which could be adjusted to left or right-handed polarization depending on the chirality of the biological target.

## 10. CONCLUSION

IDPs exhibit exceptional sensitivity to their hydration environments, which, under structured conditions, may exhibit quantum-coherent properties. Aging and environmental stressors—such as oxidative damage and posttranslational modifications (PTMs)—can destabilize this finely tuned protein-water interface. Such disruptions may contribute to hallmark features of aging and neurodegeneration by impairing the dynamic conformational landscape of IDPs. This shift can lead to a loss of PTM-mediated functional plasticity, promote pathological aggregation, or hinder essential protein-DNA interactions, as observed in age-related dysfunction of HSF1 and increased receptor binding of A*β*.

In this paper, we propose a quantum biological framework for a novel therapeutic strategy (REMFS) that can influence structured water, proton tunneling, and charge separation. This strategy aims to restore the conformational flexibility of IDPs to a physiologically stable, non-aggregated conformation in multiple proteins, such as A*β*, tau, *α*-synuclein, and Tau, which are central to neurodegenerative disorders. Furthermore, REMFS reverses PTMs that impair the normal function of IDPs, such as HSF1 and autophagy proteins, resulting in improved protein degradation, delayed aging [165], and potentially beneficial as an anti-amyloid therapy for human AD and neurodegenerative diseases [264]. By stabilizing hydration shells and reestablishing quantum coherence, EMFs may act as biophysical regulators of proteostasis. This insight introduces a paradigm shift: hydration shells are not passive solvents but active quantum interfaces that govern protein behavior. A recently developed human REMFS (repeated electromagnetic field stimulation) device offers a translational pathway for clinical application. Future iterations should incorporate harmonic frequency tuning and spatial field shaping to enable targeted modulation of IDPs based on their unique biophysical signatures. Such precision could enhance therapeutic efficacy while minimizing off-target effects.

Ultimately, this approach may enable fine-tuned control over protein conformation and signaling pathways by targeting structured water scaffolds. A novel design and manufacture of an appropriate human REMFS device was recently introduced as a potential pathway for clinical translation [264]. Building on this foundation, future iterations should consider the integration of harmonic frequency controls and field shaping capabilities, enabling targeted stimulation based on the specific biophysical properties of IDPs. This could enhance selectivity and therapeutic efficacy while minimizing off-target effects [265, 266]. This may someday fine-tune protein conformation and molecular pathway activation by targeting the structured water scaffolds that will make it possible to prevent and reverse AD, and other neurodegenerative diseases associated with protein aggregation such as Parkinson’s, Lewy body, Frontotemporal, and Multisystem Atrophy.

## References

[1] Kinsey LJ, Beane WS and Tseng KA (2024) Accelerating an Integrative View of Quantum Biology. Frontiers in Physiology, 14, Article ID: 1349013. 10.3389/fphys.2023.1349013 PMC10811782 38283282

[2] Patwa H, Babcock NS and Kurian P (2024) Quantum-Enhanced Photoprotection in Neuroprotein Architectures Emerges from Collective Light-Matter Interactions. Frontiers in Physics, 12, Article ID: 1387271. 10.3389/fphy.2024.1387271

[3] Otten M, (2024) Quantum Resources Required for Binding Affinity Calculations of Amyloid Beta.

[4] Messori C, Prinzera SV and di Bardone FB (2019) Deep into the Water: Exploring the Hydro-Electromagnetic and Quantum-Electrodynamic Properties of Interfacial Water in Living Systems. Open Access Library Journal, 6, 1–50. 10.4236/oalib.1105435

[5] Chen L, Zhang S, Liu X and Ge X (2023) Recent Advances in Water-Mediated Multiphase Catalysis. Current Opinion in Colloid & Interface Science, 65, Article ID: 101691. 10.1016/j.cocis.2023.101691

[6] Reid KM, Singh AK, Bikash CR, Wei J, Tal-Gan Y, Vinh NQ, (2022) The Origin and Impact of Bound Water around Intrinsically Disordered Proteins. Biophysical Journal, 121, 540–551. 10.1016/j.bpj.2022.01.011 35074392 PMC8874019

[7] Assaker K, Carteret C, Lebeau B, Marichal C, Vidal L, Stébé M, (2013) Water-Catalyzed Low-Temperature Transformation from Amorphous to Semi-Crystalline Phase of Ordered Mesoporous Titania Framework. ACS Sustainable Chemistry & Engineering, 2, 120–125. 10.1021/sc400323w

[8] Giudice ED, Tedeschi A, Vitiello G and Voeikov V (2013) Coherent Structures in Liquid Water Close to Hydrophilic Surfaces. Journal of Physics: Conference Series, 442, Article ID: 012028. 10.1088/1742-6596/442/1/012028

[9] Peng Z, Yan J, Fan X, Mizianty MJ, Xue B, Wang K, (2014) Exceptionally Abundant Exceptions: Comprehensive Characterization of Intrinsic Disorder in All Domains of Life. Cellular and Molecular Life Sciences, 72, 137–151. 10.1007/s00018-014-1661-9 24939692 PMC11113594

[10] Stavropoulos I, Khaldi N, Davey NE, O’Brien K, Martin F and Shields DC (2012) Protein Disorder and Short Conserved Motifs in Disordered Regions Are Enriched near the Cytoplasmic Side of Single-Pass Transmembrane Proteins. PLOS ONE, 7, e44389. 10.1371/journal.pone.0044389 22962613 PMC3433447

[11] Uversky VN (2009) Intrinsically Disordered Proteins and Their Environment: Effects of Strong Denaturants, Temperature, pH, Counter Ions, Membranes, Binding Partners, Osmolytes, and Macromolecular Crowding. The Protein Journal, 28, 305–325. 10.1007/s10930-009-9201-4 19768526

[12] Uversky VN (2011) Intrinsically Disordered Proteins from a to Z. The International Journal of Biochemistry & Cell Biology, 43, 1090–1103. 10.1016/j.biocel.2011.04.001 21501695

[13] Uversky VN, Li J and Fink AL (2001) Evidence for a Partially Folded Intermediate in *α*-Synuclein Fibril Formation. Journal of Biological Chemistry, 276, 10737–10744. 10.1074/jbc.m010907200 11152691

[14] Ruff KM, Roberts S, Chilkoti A and Pappu RV (2018) Advances in Understanding Stimulus-Responsive Phase Behavior of Intrinsically Disordered Protein Polymers. Journal of Molecular Biology, 430, 4619–4635. 10.1016/j.jmb.2018.06.031 29949750

[15] Fonin AV, Darling AL, Kuznetsova IM, Turoverov KK and Uversky VN (2018) Intrinsically Disordered Proteins in Crowded Milieu: When Chaos Prevails within the Cellular Gumbo. Cellular and Molecular Life Sciences, 75, 3907–3929. 10.1007/s00018-018-2894-9 30066087 PMC11105604

[16] Turoverov KK, Kuznetsova IM and Uversky VN (2010) The Protein Kingdom Extended: Ordered and Intrinsically Disordered Proteins, Their Folding, Supramolecular Complex Formation, and Aggregation. Progress in Biophysics and Molecular Biology, 102, 73–84. 10.1016/j.pbiomolbio.2010.01.003 20097220 PMC2916636

[17] Dogan J, Gianni S and Jemth P (2014) The Binding Mechanisms of Intrinsically Disordered Proteins. Physical Chemistry Chemical Physics, 16, 6323–6331. 10.1039/c3cp54226b 24317797

[18] Wright PE and Dyson HJ (2014) Intrinsically Disordered Proteins in Cellular Signalling and Regulation. Nature Reviews Molecular Cell Biology, 16, 18–29. 10.1038/nrm3920 PMC4405151 25531225

[19] Fisher CK and Stultz CM (2011) Constructing Ensembles for Intrinsically Disordered Proteins. Current Opinion in Structural Biology, 21, 426–431. 10.1016/j.sbi.2011.04.001 21530234 PMC3112268

[20] Uversky VN (2013) Unusual Biophysics of Intrinsically Disordered Proteins. Biochimica et Biophysica Acta (BBA)—Proteins and Proteomics, 1834, 932–951. 10.1016/j.bbapap.2012.12.008 23269364

[21] van der Lee R, Buljan M, Lang B, Weatheritt RJ, Daughdrill GW, Dunker AK, (2014) Classification of Intrinsically Disordered Regions and Proteins. Chemical Reviews, 114, 6589–6631. 10.1021/cr400525m 24773235 PMC4095912

[22] Darling AL and Uversky VN (2018) Intrinsic Disorder and Posttranslational Modifications: The Darker Side of the Biological Dark Matter. Frontiers in Genetics, 9, Article No. 158. 10.3389/fgene.2018.00158 PMC5945825 29780404

[23] Mann M and Jensen ON (2003) Proteomic Analysis of Post-Translational Modifications. Nature Biotechnology, 21, 255–261. 10.1038/nbt0303-255 12610572

[24] Youle RJ and Strasser A (2008) The BCL-2 Protein Family: Opposing Activities That Mediate Cell Death. Nature Reviews Molecular Cell Biology, 9, 47–59. 10.1038/nrm2308 18097445

[25] Jungblut PR, Holzhütter HG, Apweiler R and Schlüter H (2008) The Speciation of the Proteome. Chemistry Central Journal, 2, 1–10. 10.1186/1752-153x-2-16 18638390 PMC2492845

[26] Kulkarni P, Jolly MK, Jia D, Mooney SM, Bhargava A, Kagohara LT, (2017) Phosphorylation-Induced Conformational Dynamics in an Intrinsically Disordered Protein and Potential Role in Phenotypic Heterogeneity. Proceedings of the National Academy of Sciences, 114, E2644–E2653. 10.1073/pnas.1700082114 PMC5380051 28289210

[27] Saito M, Hess D, Eglinger J, Fritsch AW, Kreysing M, Weinert BT, (2018) Acetylation of Intrinsically Disordered Regions Regulates Phase Separation. Nature Chemical Biology, 15, 51–61. 10.1038/s41589-018-0180-7 30531905

[28] Rahman MM, Zamakhaeva S, Rush JS, Chaton CT, Kenner CW, Hla YM, (2025) Glycosylation of Serine/Threonine-Rich Intrinsically Disordered Regions of Membrane-Associated Proteins in Streptococci. Nature Communications, 16, Article No. 4011. 10.1038/s41467-025-58692-8 PMC12041528 40301326

[29] Zhang Z, Ji J, Hossain MS, Bailey B, Nangia S and Mozhdehi D (2024) Lipidation Alters the Phase-Separation of Resilin-Like Polypeptides. Soft Matter, 20, 4007–4014. 10.1039/d4sm00358f 38690757 PMC11095499

[30] Bauer V, Schmidtgall B, Gógl G, Dolenc J, Osz J, Nominé Y, (2021) Conformational Editing of Intrinsically Disordered Protein by *α*-Methylation. Chemical Science, 12, 1080–1089. 10.1039/d0sc04482b PMC8178997 34163874

[31] Uversky VN, Yamin G, Munishkina LA, Karymov MA, Millett IS, Doniach S, (2005) Effects of Nitration on the Structure and Aggregation of *α*-Synuclein. Molecular Brain Research, 134, 84–102. 10.1016/j.molbrainres.2004.11.014 15790533

[32] Geist L, Henen MA, Haiderer S, Schwarz TC, Kurzbach D, Zawadzka-Kazimierczuk A, (2013) Protonation-Dependent Conformational Variability of Intrinsically Disordered Proteins. Protein Science, 22, 1196–1205. 10.1002/pro.2304 23821606 PMC3776332

[33] Feng J, She Y, Li C and Shen L (2023) Metal Ion Mediated Aggregation of Alzheimer’s Disease Peptides and Proteins in Solutions and at Surfaces. Advances in Colloid and Interface Science, 320, Article ID: 103009. 10.1016/j.cis.2023.103009 37776735

[34] Wise-Scira O, Dunn A, Aloglu AK, Sakallioglu IT and Coskuner O (2013) Structures of the E46K Mutant-Type *α*-Synuclein Protein and Impact of E46K Mutation on the Structures of the Wild-Type *α*-Synuclein Protein. ACS Chemical Neuroscience, 4, 498–508. 10.1021/cn3002027 23374074 PMC3605821

[35] Levine ZA, Larini L, LaPointe NE, Feinstein SC and Shea J (2015) Regulation and Aggregation of Intrinsically Disordered Peptides. Proceedings of the National Academy of Sciences, 112, 2758–2763. 10.1073/pnas.1418155112 PMC4352815 25691742

[36] Kjaergaard M, Nørholm A, Hendus‒Altenburger R, Pedersen SF, Poulsen FM and Kragelund BB (2010) Temperature-Dependent Structural Changes in Intrinsically Disordered Proteins: Formation of *α*-Helices or Loss of Polyproline II? Protein Science, 19, 1555–1564. 10.1002/pro.435 20556825 PMC2923508

[37] Vidović M and Komić Milić S (2021) Regulation of Proteolysis of Intrinsically Disordered Proteins: Physiological Consequences. A Closer Look at Proteolysis. 1–46.

[38] Uversky VN, Oldfield CJ and Dunker AK (2008) Intrinsically Disordered Proteins in Human Diseases: Introducing the D^2^ Concept. Annual Review of Biophysics, 37, 215–246. 10.1146/annurev.biophys.37.032807.125924 18573080

[39] Tanaka M, Morita S and Hayashi T (2021) Role of Interfacial Water in Determining the Interactions of Proteins and Cells with Hydrated Materials. Colloids and Surfaces B: Biointerfaces, 198, Article ID: 111449. 10.1016/j.colsurfb.2020.111449 33310639

[40] Seneff S and Kyriakopoulos AM (2025) Taurine Prevents Mitochondrial Dysfunction and Protects Mitochondria from Reactive Oxygen Species and Deuterium Toxicity. Amino Acids, 57, Article No. 6. 10.1007/s00726-024-03440-3 PMC11717795 39789296

[41] Figueroa XA and Pollack GH (2011) Exclusion-Zone Formation from Discontinuous Nafion Surfaces. International Journal of Design & Nature and Ecodynamics, 6, 286–296. 10.2495/dne-v6-n4-286-296 PMC4016955 24826197

[42] Elton DC, Spencer PD, Riches JD and Williams ED (2020) Exclusion Zone Phenomena in Water—A Critical Review of Experimental Findings and Theories. International Journal of Molecular Sciences, 21, Article No. 5041. 10.3390/ijms21145041 PMC7404113 32708867

[43] Lin L, Jiang W, Xu X and Xu P (2020) A Critical Review of the Application of Electromagnetic Fields for Scaling Control in Water Systems: Mechanisms, Characterization, and Operation. NPJ Clean Water, 3, Article No. 25. 10.1038/s41545-020-0071-9

[44] Chai B, Mahtani AG and Pollack GH (2012) Unexpected Presence of Solute-Free Zones at Metal-Water Interfaces. Contemporary Materials, 3, 1–12. 10.7251/com1201001c 23807904 PMC3692373

[45] Chai B, Yoo H and Pollack GH (2009) Effect of Radiant Energy on Near-Surface Water. The Journal of Physical Chemistry B, 113, 13953–13958. 10.1021/jp908163w 19827846 PMC2843558

[46] Rad I, Stahlberg R, Kung K and Pollack GH (2021) Low Frequency Weak Electric Fields Can Induce Structural Changes in Water. PLOS ONE, 16, e0260967. 10.1371/journal.pone.0260967 34855917 PMC8639071

[47] Del Giudice E, Preparata G and Vitiello G (1988) Water as a Free Electric Dipole Laser. Physical Review Letters, 61, 1085–1088. 10.1103/physrevlett.61.1085 10039515

[48] De Ninno A, Del Giudice E, Gamberale L and Castellano AC (2013) The Structure of Liquid Water Emerging from the Vibrational Spectroscopy: Interpretation with QED Theory.

[49] Pollack GH (2019) The Fourth Phase of Water. Tantor Audio.

[50] Taschin A, Bartolini P, Eramo R, Righini R and Torre R (2013) Evidence of Two Distinct Local Structures of Water from Ambient to Supercooled Conditions. Nature Communications, 4, Article No. 2401. 10.1038/ncomms3401 24029922

[51] Geesink HJ, Jerman I and Meijer DK (2020) Water, the Cradle of Life via Its Coherent Quantum Frequencies. Water, 11, 78–108.

[52] Ho M (2015) Illuminating Water and Life: Emilio Del Giudice. Electromagnetic Biology and Medicine, 34, 113–122. 10.3109/15368378.2015.1036079 26098522

[53] Otting G, Liepinsh E and Wüthrich K (1991) Protein Hydration in Aqueous Solution. Science, 254, 974–980. 10.1126/science.1948083 1948083

[54] Moilanen DE, Piletic IR and Fayer MD (2007) Water Dynamics in Nafion Fuel Cell Membranes: The Effects of Confinement and Structural Changes on the Hydrogen Bond Network. The Journal of Physical Chemistry C, 111, 8884–8891. 10.1021/jp067460k PMC2523265 18728757

[55] Mentré P (2004) Interfacial Water: A Modulator of Biological Activity. Journal of Biological Physics and Chemistry, 4, 115–123. 10.4024/10me04r.jbpc.04.02

[56] Arunan E, Desiraju GR, Klein RA, Sadlej J, Scheiner S, Alkorta I, (2011) Defining the Hydrogen Bond: An Account (IUPAC Technical Report). Pure and Applied Chemistry, 83, 1619–1636. 10.1351/pac-rep-10-01-01

[57] Ball P (2007) Water as an Active Constituent in Cell Biology. Chemical Reviews, 108, 74–108. 10.1021/cr068037a 18095715

[58] Pal SK and Zewail AH (2004) Dynamics of Water in Biological Recognition. Chemical Reviews, 104, 2099–2124. 10.1021/cr020689l 15080722

[59] Nguyen TH, Zhang C, Weichselbaum E, Knyazev DG, Pohl P and Carloni P (2018) Interfacial Water Molecules at Biological Membranes: Structural Features and Role for Lateral Proton Diffusion. PLOS ONE, 13, e0193454. 10.1371/journal.pone.0193454 29474432 PMC5825111

[60] Bothma JP, Gilmore JB and McKenzie RH (2010) The Role of Quantum Effects in Proton Transfer Reactions in Enzymes: Quantum Tunneling in a Noisy Environment? New Journal of Physics, 12, Article ID: 055002. 10.1088/1367-2630/12/5/055002

[61] Meng X, Guo J, Peng J, Chen J, Wang Z, Shi J, (2015) Direct Visualization of Concerted Proton Tunnelling in a Water Nanocluster. Nature Physics, 11, 235–239. 10.1038/nphys3225

[62] Allemann RK and Scrutton NS (2009) Quantum Tunnelling in Enzyme-Catalysed Reactions. Royal Society of Chemistry.

[63] del Giudice E, Doglia S, Milani M and Vitiello G (1986) Electromagnetic Field and Spontaneous Symmetry Breaking in Biological Matter. Nuclear Physics B, 275, 185–199. 10.1016/0550-3213(86)90595-x

[64] Fichou Y, Schirò G, Gallat F, Laguri C, Moulin M, Combet J, (2015) Hydration Water Mobility Is Enhanced around Tau Amyloid Fibers. Proceedings of the National Academy of Sciences, 112, 6365–6370. 10.1073/pnas.1422824112 PMC4443308 25918405

[65] Camino JD, Gracia P and Cremades N (2021) The Role of Water in the Primary Nucleation of Protein Amyloid Aggregation. Biophysical Chemistry, 269, Article ID: 106520. 10.1016/j.bpc.2020.106520 33341693

[66] Bellissent-Funel M, Hassanali A, Havenith M, Henchman R, Pohl P, Sterpone F, (2016) Water Determines the Structure and Dynamics of Proteins. Chemical Reviews, 116, 7673–7697. 10.1021/acs.chemrev.5b00664 27186992 PMC7116073

[67] Elgabarty H, Kaliannan NK and Kühne TD (2019) Enhancement of the Local Asymmetry in the Hydrogen Bond Network of Liquid Water by an Ultrafast Electric Field Pulse. Scientific Reports, 9, Article No. 10002. 10.1038/s41598-019-46449-5 PMC6620291 31292493

[68] Zhao H, Tan Y, Zhang L, Zhang R, Shalaby M, Zhang C, (2020) Ultrafast Hydrogen Bond Dynamics of Liquid Water Revealed by Terahertz-Induced Transient Birefringence. Light: Science & Applications, 9, 1–10. 10.1038/s41377-020-00370-z PMC7403349 32802323

[69] Faupin J, Fröhlich J and Schubnel B (2015) On the Probabilistic Nature of Quantum Mechanics and the Notion of Closed Systems. Annales Henri Poincaré, 17, 689–731. 10.1007/s00023-015-0416-y

[70] Day TJF, Schmitt UW and Voth GA (2000) The Mechanism of Hydrated Proton Transport in Water. Journal of the American Chemical Society, 122, 12027–12028. 10.1021/ja002506n

[71] Siwick BJ and Bakker HJ (2007) On the Role of Water in Intermolecular Proton-Transfer Reactions. Journal of the American Chemical Society, 129, 13412–13420. 10.1021/ja069265p 17935322

[72] Odutola JA, Hu TA, Prinslow D, O’dell SE and Dyke TR (1988) Water Dimer Tunneling States with *k* = 0. The Journal of Chemical Physics, 88, 5352–5361. 10.1063/1.454595

[73] Rao ML, Sedlmayr SR, Roy R and Kanzius J (2010) Polarized Microwave and RF Radiation Effects on the Structure and Stability of Liquid Water. Current Science, 98, 1500–1504.

[74] Vallée P, Lafait J, Legrand L, Mentré P, Monod M and Thomas Y (2005) Effects of Pulsed Low-Frequency Electromagnetic Fields on Water Characterized by Light Scattering Techniques: Role of Bubbles. Langmuir, 21, 2293–2299. 10.1021/la047916u 15752018

[75] Calabrò E and Magazù S (2017) Response of Hydrogen Bonding to Low-Intensity 50 Hz Electromagnetic Field in Typical Proteins in Bi-Distilled Water Solution. Spectroscopy Letters, 50, 330–335. 10.1080/00387010.2017.1328444

[76] James D and Armishaw R (1975) Structure of Aqueous Solutions: Infrared Spectra of the Water Librational Mode in Solutions of Monovalent Halides. Australian Journal of Chemistry, 28, 1179–1186. 10.1071/ch9751179

[77] English NJ and MacElroy JMD (2003) Molecular Dynamics Simulations of Microwave Heating of Water. The Journal of Chemical Physics, 118, 1589–1592. 10.1063/1.1538595

[78] Panagopoulos DJ, Messini N, Karabarbounis A, Philippetis AL and Margaritis LH (2000) A Mechanism for Action of Oscillating Electric Fields on Cells. Biochemical and Biophysical Research Communications, 272, 634–640. 10.1006/bbrc.2000.2746 10860806

[79] Panagopoulos DJ, Johansson O and Carlo GL (2015) Polarization: A Key Difference between Man-Made and Natural Electromagnetic Fields, in Regard to Biological Activity. Scientific Reports, 5, Article No. 14914. 10.1038/srep14914 PMC4601073 26456585

[80] Philbin TG (2012) Quantum Dynamics of the Damped Harmonic Oscillator. New Journal of Physics, 14, Article ID: 083043. 10.1088/1367-2630/14/8/083043

[81] Pikovsky A, Rosenblum M and Kurths J (2001) Synchronization: A Unified Approach to Nonlinear Science. Cambridge University Press. 10.1017/cbo9780511755743

[82] Samdal S (1994) The Effect of Large Amplitude Motion on the Comparison of Bond Distances from *Ab Initio* Calculations and Experimentally Determined Bond Distances, and on Root-Mean-Square Amplitudes of Vibration, Shrinkage, Asymmetry Constants, Symmetry Constraints, and Inclusion of Rotational Constants Using the Electron Diffraction Method. Journal of Molecular Structure, 318, 133–141. 10.1016/0022-2860(93)07897-6

[83] Kirillova S and Carugo O (2011) Hydration Sites of Unpaired RNA Bases: A Statistical Analysis of the PDB Structures. BMC Structural Biology, 11, Article No. 41. 10.1186/1472-6807-11-41 PMC3206426 22011380

[84] Grifoni M and Hänggi P (1998) Driven Quantum Tunneling. Physics Reports, 304, 229–354. 10.1016/s0370-1573(98)00022-2

[85] Shi S, Zhang Q, Zhang L, Wang R, Zhu Z, Jiang G, (2011) Geometrical Structures, Vibrational Frequencies, Force Constants and Dissociation Energies of Isotopic Water Molecules (H_2_O, HDO, D_2_O, HTO, DTO, and T_2_O) under Dipole Electric Field. Chinese Physics B, 20, Article ID: 063102. 10.1088/1674-1056/20/6/063102

[86] Baranyai A, Bartók A and Chialvo AA (2005) Computer Simulation of the 13 Crystalline Phases of Ice. The Journal of Chemical Physics, 123, Article ID: 054502. 10.1063/1.1989313 16108664

[87] Laage D and Hynes JT (2006) A Molecular Jump Mechanism of Water Reorientation. Science, 311, 832–835. 10.1126/science.1122154 16439623

[88] Marx D, Tuckerman ME, Hutter J and Parrinello M (1999) The Nature of the Hydrated Excess Proton in Water. Nature, 397, 601–604. 10.1038/17579

[89] Bagchi B (2005) Water Dynamics in the Hydration Layer around Proteins and Micelles. Chemical Reviews, 105, 3197–3219. 10.1021/cr020661+ 16159150

[90] Schönichen A, Webb BA, Jacobson MP and Barber DL (2013) Considering Protonation as a Posttranslational Modification Regulating Protein Structure and Function. Annual Review of Biophysics, 42, 289–314. 10.1146/annurev-biophys-050511-102349 PMC4041481 23451893

[91] Fossat MJ (2025) MEDOC: A Fast, Scalable, and Mathematically Exact Algorithm for the Site-Specific Prediction of the Protonation Degree in Large Disordered Proteins. Journal of Chemical Information and Modeling, 65, 873–881. 10.1021/acs.jcim.4c01860 39817437 PMC11776046

[92] Pelton JG, Torchia DA, Meadow ND and Roseman S (1993) Tautomeric States of the Active-Site Histidines of Phosphorylated and Unphosphorylated III^glc^, a Signal-Transducing Protein from *Escherichia coli*, Using Two-dimensional Heteronuclear NMR Techniques. Protein Science, 2, 543–558. 10.1002/pro.5560020406 8518729 PMC2142369

[93] Cerón-Carrasco JP and Jacquemin D (2013) Electric-Field Induced Mutation of DNA: A Theoretical Investigation of the GC Base Pair. Physical Chemistry Chemical Physics, 15, 4548–4553. 10.1039/c2cp44066k 23338206

[94] Cerón-Carrasco JP and Jacquemin D (2013) Electric Field Induced DNA Damage: An Open Door for Selective Mutations. Chemical Communications, 49, 7578–7580. 10.1039/c3cc42593b 23722648

[95] Cerón-Carrasco JP, Cerezo J and Jacquemin D (2014) How DNA Is Damaged by External Electric Fields: Selective Mutation vs. Random Degradation. Physical Chemistry Chemical Physics, 16, 8243–8246. 10.1039/c3cp54518k 24343518

[96] Antonov L (2016) Tautomerism: Concepts and Applications in Science and Technology. John Wiley & Sons.

[97] Singh V, Fedeles BI and Essigmann JM (2014) Role of Tautomerism in RNA Biochemistry. RNA, 21, 1–13. 10.1261/rna.048371.114 PMC4274630 25516996

[98] Abou-Zied OK, Jimenez R and Romesberg FE (2001) Tautomerization Dynamics of a Model Base Pair in DNA. Journal of the American Chemical Society, 123, 4613–4614. 10.1021/ja003647s 11457253

[99] Peng CS, Jones KC and Tokmakoff A (2011) Anharmonic Vibrational Modes of Nucleic Acid Bases Revealed by 2D IR Spectroscopy. Journal of the American Chemical Society, 133, 15650–15660. 10.1021/ja205636h 21861514

[100] Aggarwal L and Biswas P (2018) Hydration Water Distribution around Intrinsically Disordered Proteins. The Journal of Physical Chemistry B, 122, 4206–4218. 10.1021/acs.jpcb.7b11091 29553735

[101] Grubmüller H, Heller H, Windemuth A and Schulten K (1991) Generalized Verlet Algorithm for Efficient Molecular Dynamics Simulations with Long-Range Interactions. Molecular Simulation, 6, 121–142. 10.1080/08927029108022142

[102] Maity H, Baidya L and Reddy G (2022) Salt-Induced Transitions in the Conformational Ensembles of Intrinsically Disordered Proteins. The Journal of Physical Chemistry B, 126, 5959–5971. 10.1021/acs.jpcb.2c03476 35944496

[103] Kulkarni P, Bhattacharya S, Achuthan S, Behal A, Jolly MK, Kotnala S, (2022) Intrinsically Disordered Proteins: Critical Components of the Wetware. Chemical Reviews, 122, 6614–6633. 10.1021/acs.chemrev.1c00848 35170314 PMC9250291

[104] Zhang T, Faraggi E, Li Z and Zhou Y (2013) Intrinsically Semi-Disordered State and Its Role in Induced Folding and Protein Aggregation. Cell Biochemistry and Biophysics, 67, 1193–1205. 10.1007/s12013-013-9638-0 23723000 PMC3838602

[105] Nandi PK, Futera Z and English NJ (2016) Perturbation of Hydration Layer in Solvated Proteins by External Electric and Electromagnetic Fields: Insights from Non-Equilibrium Molecular Dynamics. The Journal of Chemical Physics, 145, Article ID: 205101. 10.1063/1.4967774 27908109

[106] Upadhyay A and Ekenna C (2023) A New Tool to Study the Binding Behavior of Intrinsically Disordered Proteins. International Journal of Molecular Sciences, 24, Article No. 11785. 10.3390/ijms241411785 PMC10380747 37511544

[107] Abyzov A, Blackledge M and Zweckstetter M (2022) Conformational Dynamics of Intrinsically Disordered Proteins Regulate Biomolecular Condensate Chemistry. Chemical Reviews, 122, 6719–6748. 10.1021/acs.chemrev.1c00774 35179885 PMC8949871

[108] Kolesnikov AI, Reiter GF, Choudhury N, Prisk TR, Mamontov E, Podlesnyak A, (2016) Quantum Tunneling of Water in Beryl: A New State of the Water Molecule. Physical Review Letters, 116, Article ID: 167802. 10.1103/physrevlett.116.167802 27152824

[109] Mao AH, Lyle N and Pappu RV (2012) Describing Sequence-Ensemble Relationships for Intrinsically Disordered Proteins. Biochemical Journal, 449, 307–318. 10.1042/bj20121346 PMC4074364 23240611

[110] Mitrea DM and Kriwacki RW (2016) Phase Separation in Biology; Functional Organization of a Higher Order. Cell Communication and Signaling, 14, 1–20. 10.1186/s12964-015-0125-7 26727894 PMC4700675

[111] Kato M, Han TW, Xie S, Shi K, Du X, Wu LC, (2012) Cell-Free Formation of RNA Granules: Low Complexity Sequence Domains Form Dynamic Fibers within Hydrogels. Cell, 149, 753–767. 10.1016/j.cell.2012.04.017 22579281 PMC6347373

[112] Dormann D and Lemke EA (2024) Adding Intrinsically Disordered Proteins to Biological Ageing Clocks. Nature Cell Biology, 26, 851–858. 10.1038/s41556-024-01423-w 38783141

[113] Manyilov VD, Ilyinsky NS, Nesterov SV, Saqr BMGA, Dayhoff GW, Zinovev EV, (2023) Chaotic Aging: Intrinsically Disordered Proteins in Aging-Related Processes. Cellular and Molecular Life Sciences, 80, Article No. 269. 10.1007/s00018-023-04897-3 PMC11073068 37634152

[114] López-Otín C, Blasco MA, Partridge L, Serrano M and Kroemer G (2023) Hallmarks of Aging: An Expanding Universe. Cell, 186, 243–278. 10.1016/j.cell.2022.11.001 36599349

[115] Martinelli AHS, Lopes FC, John EBO, Carlini CR and Ligabue-Braun R (2019) Modulation of Disordered Proteins with a Focus on Neurodegenerative Diseases and Other Pathologies. International Journal of Molecular Sciences, 20, Article No. 1322. 10.3390/ijms20061322 PMC6471803 30875980

[116] Anbo H, Sato M, Okoshi A and Fukuchi S (2019) Functional Segments on Intrinsically Disordered Regions in Disease-Related Proteins. Biomolecules, 9, Article No. 88. 10.3390/biom9030088 PMC6468909 30841624

[117] Scholes GD, (2017) Using Coherence to Enhance Function in Chemical and Biophysical Systems. Nature (London), 543, 647–656. 10.1038/nature21425 28358065

[118] Bennett CH and DiVincenzo DP (2000) Quantum Information and Computation. Nature, 404, 247–255. 10.1038/35005001 10749200

[119] Cavanagh J, Fairbrother WJ, Palmer AG, Rance M and Skelton NJ (2007) Heteronuclear NMR Experiments. In: Cavanagh J, Fairbrother WJ, Palmer AG, Rance M and Skelton NJ, Eds., Protein NMR Spectroscopy, Elsevier, 533–678. 10.1016/b978-012164491-8/50009-9

[120] Kragelj J, Ozenne V, Blackledge M and Jensen MR (2013) Conformational Propensities of Intrinsically Disordered Proteins from NMR Chemical Shifts. ChemPhysChem, 14, 3034–3045. 10.1002/cphc.201300387 23794453

[121] Narth C, Gillet N, Cailliez F, Lévy B and de la Lande A (2015) Electron Transfer, Decoherence, and Protein Dynamics: Insights from Atomistic Simulations. Accounts of Chemical Research, 48, 1090–1097. 10.1021/ar5002796 25730126

[122] Tenenbaum A (2021) Kinetic Coherence Underlies the Dynamics of Disordered Proteins. RSC Advances, 11, 36242–36249. 10.1039/d1ra06823g 35492753 PMC9043365

[123] Rather SR, Scholes GD and Chen LX (2024) From Coherence to Function: Exploring the Connection in Chemical Systems. Accounts of Chemical Research, 57, 2620–2630. 10.1021/acs.accounts.4c00312 39222721

[124] Binolfi A, Theillet F and Selenko P (2012) Bacterial In-Cell NMR of Human A-Synuclein: A Disordered Monomer by Nature? Biochemical Society Transactions, 40, 950–954. 10.1042/bst20120096 22988846

[125] Usselman RJ, Chavarriaga C, Castello PR, Procopio M, Ritz T, Dratz EA, (2016) The Quantum Biology of Reactive Oxygen Species Partitioning Impacts Cellular Bioenergetics. Scientific Reports, 6, Article No. 38543. 10.1038/srep38543 PMC5172244 27995996

[126] Pesce F, Bremer A, Tesei G, Hopkins JB, Grace CR, Mittag T, (2024) Design of Intrinsically Disordered Protein Variants with Diverse Structural Properties. Science Advances, 10, eadm9926. 10.1126/sciadv.adm9926 39196930 PMC11352843

[127] Gehi BR, Gadhave K, Uversky VN and Giri R (2022) Intrinsic Disorder in Proteins Associated with Oxidative Stress-Induced JNK Signaling. Cellular and Molecular Life Sciences, 79, Article No. 202. 10.1007/s00018-022-04230-4 PMC11073203 35325330

[128] Leterrier J (2001) Water and the Cytoskeleton. Cellular and Molecular Biology (Noisy-le-Grand, France), 47, 901–923. 11728102

[129] Westerheide SD and Morimoto RI (2005) Heat Shock Response Modulators as Therapeutic Tools for Diseases of Protein Conformation. Journal of Biological Chemistry, 280, 33097–33100. 10.1074/jbc.r500010200 16076838

[130] Gomez-Pastor R, Burchfiel ET and Thiele DJ (2017) Regulation of Heat Shock Transcription Factors and Their Roles in Physiology and Disease. Nature Reviews Molecular Cell Biology, 19, 4–19. 10.1038/nrm.2017.73 28852220 PMC5794010

[131] Sengupta U and Kayed R (2022) Amyloid B, Tau, and *α*-Synuclein Aggregates in the Pathogenesis, Prognosis, and Therapeutics for Neurodegenerative Diseases. Progress in Neurobiology, 214, Article ID: 102270. 10.1016/j.pneurobio.2022.102270 35447272

[132] Firman T and Ghosh K (2017) Sequence Charge Decoration Dictates Coil-Globule Transition in Intrinsically Disordered Proteins. The Journal of Chemical Physics, 148, Article ID: 123305. 10.1063/1.5005821 29604827

[133] Theillet F, Binolfi A, Frembgen-Kesner T, Hingorani K, Sarkar M, Kyne C, (2014) Physicochemical Properties of Cells and Their Effects on Intrinsically Disordered Proteins (IDPs). Chemical Reviews, 114, 6661–6714. 10.1021/cr400695p 24901537 PMC4095937

[134] Gao J and Xu D (2012) Correlation between Posttranslational Modification and Intrinsic Disorder in Protein. Pacific Symposium on Biocomputing, 94–103. 10.1142/9789814366496_0010 22174266 PMC5120255

[135] Khoury GA, Baliban RC and Floudas CA (2011) Proteome-Wide Post-Translational Modification Statistics: Frequency Analysis and Curation of the Swiss-Prot Database. Scientific Reports, 1, Article No. 90. 10.1038/srep00090 PMC3201773 22034591

[136] Zhao B, Katuwawala A, Oldfield CJ, Hu G, Wu Z, Uversky VN, (2021) Intrinsic Disorder in Human RNA-Binding Proteins. Journal of Molecular Biology, 433, Article ID: 167229. 10.1016/j.jmb.2021.167229 34487791

[137] Liu AY, Minetti CA, Remeta DP, Breslauer KJ and Chen KY (2022) HSF1, Aging, and Neurodegeneration. In: Turksen K, Ed., Cell Biology and Translational Medicine, Volume 18: Tissue Differentiation, Repair in Health and Disease, Springer, 23–49. 10.1007/5584_2022_733 35995906

[138] Ren Q, (2025) The Molecular Mechanism of Temperature-Dependent Phase Separation of Heat Shock Factor 1. Nature Chemical Biology, 21, 831–842. 39794489 10.1038/s41589-024-01806-y

[139] Shamovsky I, Ivannikov M, Kandel ES, Gershon D and Nudler E (2006) RNA-Mediated Response to Heat Shock in Mammalian Cells. Nature, 440, 556–560. 10.1038/nature04518 16554823

[140] Zhang H, Shao S, Zeng Y, Wang X, Qin Y, Ren Q, (2022) Reversible Phase Separation of HSF1 Is Required for an Acute Transcriptional Response during Heat Shock. Nature Cell Biology, 24, 340–352. 10.1038/s41556-022-00846-7 35256776

[141] Anckar J and Sistonen L (2011) Regulation of HSF1 Function in the Heat Stress Response: Implications in Aging and Disease. Annual Review of Biochemistry, 80, 1089–1115. 10.1146/annurev-biochem-060809-095203 21417720

[142] Huang C, Wu J, Xu L, Wang J, Chen Z and Yang R (2018) Regulation of HSF1 Protein Stabilization: An Updated Review. European Journal of Pharmacology, 822, 69–77. 10.1016/j.ejphar.2018.01.005 29341886

[143] Westerheide SD, Anckar J, Stevens SM, Sistonen L and Morimoto RI (2009) Stress-Inducible Regulation of Heat Shock Factor 1 by the Deacetylase Sirt1. Science, 323, 1063–1066. 10.1126/science.1165946 19229036 PMC3429349

[144] Raynes R, Brunquell J and Westerheide SD (2013) Stress Inducibility of SIRT1 and Its Role in Cytoprotection and Cancer. Genes & Cancer, 4, 172–182. 10.1177/1947601913484497 24020008 PMC3764474

[145] Ma X, Xu L, Alberobello AT, Gavrilova O, Bagattin A, Skarulis M, (2015) Celastrol Protects against Obesity and Metabolic Dysfunction through Activation of a HSF1-PGC1*α* Transcriptional Axis. Cell Metabolism, 22, 695–708. 10.1016/j.cmet.2015.08.005 26344102

[146] Zelin E and Freeman BC (2015) Lysine Deacetylases Regulate the Heat Shock Response Including the Age-Associated Impairment of Hsf1. Journal of Molecular Biology, 427, 1644–1654. 10.1016/j.jmb.2015.02.010 25688804 PMC4357550

[147] Dasdag O, Adalier N and Dasdag S (2020) Electromagnetic Radiation and Alzheimer’s Disease. Biotechnology & Biotechnological Equipment, 34, 1087–1094. 10.1080/13102818.2020.1820378

[148] Perez FP, Bandeira JP, Perez Chumbiauca CN, Lahiri DK, Morisaki J and Rizkalla M (2022) Multidi-mensional Insights into the Repeated Electromagnetic Field Stimulation and Biosystems Interaction in Aging and Age-Related Diseases. Journal of Biomedical Science, 29, 1–22. 10.1186/s12929-022-00825-y 35698225 PMC9190166

[149] International Commission on Non-Ionizing Radiation Protection (ICNIRP) (2020) Guidelines for Limiting Exposure to Electromagnetic Fields (100 kHz to 300 GHz). Health Physics, 118, 483–524. 32167495 10.1097/HP.0000000000001210

[150] Marchesi N, Osera C, Fassina L, Amadio M, Angeletti F, Morini M, (2014) Autophagy Is Modulated in Human Neuroblastoma Cells through Direct Exposition to Low Frequency Electromagnetic Fields. Journal of Cellular Physiology, 229, 1776–1786. 10.1002/jcp.24631 24676932

[151] Hirai T, Taniura H, Goto Y, Ogura M, Sng JCG and Yoneda Y (2006) Stimulation of Ubiquitin-Proteasome Pathway through the Expression of Amidohydrolase for N-Terminal Asparagine (ntan1) in Cultured Rat Hippocampal Neurons Exposed to Static Magnetism. Journal of Neurochemistry, 96, 1519–1530. 10.1111/j.1471-4159.2006.03655.x 16539681

[152] Park J, Kwon JH, Kim N and Song K (2017) Effects of 1950 MHz Radiofrequency Electromagnetic Fields on A*β* Processing in Human Neuroblastoma and Mouse Hippocampal Neuronal Cells. Journal of Radiation Research, 59, 18–26. 10.1093/jrr/rrx045 PMC5778507 29040655

[153] Jeong Y, Kang G, Kwon J, Choi H, Pack J, Kim N, (2015) 1950 MHz Electromagnetic Fields Ameliorate A*β* Pathology in Alzheimer’s Disease Mice. Current Alzheimer Research, 12, 481–492. 10.2174/156720501205150526114448 26017559 PMC5445699

[154] Rao RR, Halper J and Kisaalita WS (2002) Effects of 60 Hz Electromagnetic Field Exposure on APP695 Transcription Levels in Differentiating Human Neuroblastoma Cells. Bioelectrochemistry, 57, 9–15. 10.1016/s1567-5394(02)00004-x 12049751

[155] Antonini RA, Benfante R, Gotti C, Moretti M, Kuster N, Schuderer J, (2006) Extremely Low-Frequency Electromagnetic Field (ELF-EMF) Does Not Affect the Expression of *α*3, *α*5 and *α*7 Nicotinic Receptor Subunit Genes in SH-SY5Y Neuroblastoma Cell Line. Toxicology Letters, 164, 268–277. 10.1016/j.toxlet.2006.01.006 16513298

[156] Del Giudice E, Facchinetti F, Nofrate V, Boccaccio P, Minelli T, Dam M, (2007) Fifty Hertz Electromagnetic Field Exposure Stimulates Secretion of *β*-Amyloid Peptide in Cultured Human Neuroglioma. Neuroscience Letters, 418, 9–12. 10.1016/j.neulet.2007.02.057 17382472

[157] He G, Luo Z, Shen T, Li P, Yang J, Luo X, (2016) Inhibition of STAT3- and MAPK-Dependent PGE2 Synthesis Ameliorates Phagocytosis of Fibrillar *β*-Amyloid Peptide (1–42) via EP2 Receptor in EMF-Stimulated N9 Microglial Cells. Journal of Neuroinflammation, 13, Article No. 296. 10.1186/s12974-016-0762-9 PMC5117690 27871289

[158] Newton TM, Duce JA and Bayle ED (2019) The Proteostasis Network Provides Targets for Neurodegeneration. British Journal of Pharmacology, 176, 3508–3514. 10.1111/bph.14643 30820936 PMC6715599

[159] Kovács D, Sigmond T, Hotzi B, Bohár B, Fazekas D, Deák V, (2019) HSF1Base: A Comprehensive Database of HSF1 (Heat Shock Factor 1) Target Genes. International Journal of Molecular Sciences, 20, Article No. 5815. 10.3390/ijms20225815 PMC6888953 31752429

[160] Perez FP, Zhou X, Morisaki J and Jurivich D (2008) Electromagnetic Field Therapy Delays Cellular Senescence and Death by Enhancement of the Heat Shock Response. Experimental Gerontology, 43, 307–316. 10.1016/j.exger.2008.01.004 18325704

[161] Manai F, (2014) A Low-Frequency Electromagnetic (LF-EMF) Exposure Scheme Induces Autophagy Activation to Counteract *in Vitro* A*β*-Amyloid Neurotoxicity. Atti del, 7.

[162] Trivedi R, Knopf B, Rakoczy S, Manocha GD, Brown-Borg H and Jurivich DA (2023) Disrupted HSF1 Regulation in Normal and Exceptional Brain Aging. Biogerontology, 25, 147–160. 10.1007/s10522-023-10063-w 37707683 PMC10794279

[163] Watanabe Y, Taguchi K and Tanaka M (2023) Roles of Stress Response in Autophagy Processes and Aging-Related Diseases. International Journal of Molecular Sciences, 24, Article No. 13804. 10.3390/ijms241813804 PMC10531041 37762105

[164] Perez P, Moinuddin FS, ul ain Shamim S, Joseph QJ, Morisaki DJ and Zhou X (2012) Longevity Pathways: HSF1 and Foxo Pathways, a New Therapeutic Target to Prevent Age-Related Diseases. Current Aging Science, 5, 87–95. 10.2174/1874609811205020087 21834787

[165] Perez FP, Zhou X, Morisaki J, Ilie J, James T and Jurivich DA (2008) Engineered Repeated Electromagnetic Field Shock Therapy for Cellular Senescence and Age-Related Diseases. Rejuvenation Research, 11, 10491058. 10.1089/rej.2008.0793 19119860

[166] Chou CK, Bassen H, Osepchuk J, Balzano Q, Petersen R, Meltz M, (1996) Radio Frequency Electromagnetic Exposure: Tutorial Review on Experimental Dosimetry. Bioelectromagnetics, 17, 195–208. 10.1002/(sici)1521-186x(1996)17:3<195::aid-bem5>3.0.co;2-z 8809359

[167] Baldi E and Bucherelli C (2005) The Inverted “u-Shaped” Dose-Effect Relationships in Learning and Memory: Modulation of Arousal and Consolidation. Nonlinearity in Biology, Toxicology, Medicine, 3, 9–21. 10.2201/nonlin.003.01.002 19330154 PMC2657842

[168] Ahmad RHMA, Fakhoury M and Lawand N (2021) Electromagnetic Field in Alzheimer’s Disease: A Literature Review of Recent Preclinical and Clinical Studies. Current Alzheimer Research, 17, 1001–1012. 10.2174/1567205017666201130085853 33256578

[169] Shirbandi K, (2023) Exposure to Low Levels of Radiofrequency Electromagnetic Fields Emitted from Cell-Phones as a Promising Treatment of Alzheimer’s Disease: A Scoping Review Study. Journal of Biomedical Physics & Engineering, 13, 3. 36818013 10.31661/jbpe.v0i0.2109-1398 PMC9923247

[170] BioInitiative (2012) A Rationale for Biologically-Based Exposure Standards for Low-Intensity Electromagnetic Radiation. 2022 Updated Research Summaries.

[171] Perez FP, Maloney B, Chopra N, Morisaki JJ and Lahiri DK (2021) Repeated Electromagnetic Field Stimulation Lowers Amyloid-*β* Peptide Levels in Primary Human Mixed Brain Tissue Cultures. Scientific Reports, 11, Article No. 621. 10.1038/s41598-020-77808-2 PMC7804462 33436686

[172] Tsoy A, Saliev T, Abzhanova E, Turgambayeva A, Kaiyrlykyzy A, Akishev M, (2019) The Effects of Mobile Phone Radiofrequency Electromagnetic Fields on *β*-Amyloid-Induced Oxidative Stress in Human and Rat Primary Astrocytes. Neuroscience, 408, 46–57. 10.1016/j.neuroscience.2019.03.058 30953670

[173] Wang C, Zhu X, Chen R, Zhang X and Lian N (2023) Upregulation of UBR1 m6A Methylation by METTL14 Inhibits Autophagy in Spinal Cord Injury. eneuro, 10, ENEURO.0338-22.2023. 10.1523/eneuro.0338-22.2023 PMC10241380 37094938

[174] Osera C, Amadio M, Falone S, Fassina L, Magenes G, Amicarelli F, (2015) Pre-Exposure of Neuroblastoma Cell Line to Pulsed Electromagnetic Field Prevents H_2_O_2_-Induced ROS Production by Increasing MnSOD Activity. Bioelectromagnetics, 36, 219–232. 10.1002/bem.21900 25708841

[175] Osera C, Fassina L, Amadio M, Venturini L, Buoso E, Magenes G, (2011) Cytoprotective Response Induced by Electromagnetic Stimulation on SH-SY5Y Human Neuroblastoma Cell Line. Tissue Engineering Part A, 17, 2573–2582. 10.1089/ten.tea.2011.0071 21615217

[176] Leszczynski D, Joenväärä S, Reivinen J and Kuokka R (2002) Non-Thermal Activation of the hsp27/p38MAPK Stress Pathway by Mobile Phone Radiation in Human Endothelial Cells: Molecular Mechanism for Cancer- and Blood-Brain Barrier-Related Effects. Differentiation, 70, 120–129. 10.1046/j.1432-0436.2002.700207.x 12076339

[177] Arendash GW, Mori T, Dorsey M, Gonzalez R, Tajiri N and Borlongan C (2012) Electromagnetic Treatment to Old Alzheimer’s Mice Reverses *β*-Amyloid Deposition, Modifies Cerebral Blood Flow, and Provides Selected Cognitive Benefit. PLOS ONE, 7, e35751. 10.1371/journal.pone.0035751 22558216 PMC3338462

[178] Arendash GW (2012) Transcranial Electromagnetic Treatment against Alzheimer’s Disease: Why It Has the Potential to Trump Alzheimer’s Disease Drug Development. Journal of Alzheimer’s Disease, 32, 243–266. 10.3233/jad-2012-120943 22810103

[179] Dragicevic N, Bradshaw PC, Mamcarz M, Lin X, Wang L, Cao C, (2011) Long-Term Electromagnetic Field Treatment Enhances Brain Mitochondrial Function of Both Alzheimer’s Transgenic Mice and Normal Mice: A Mechanism for Electromagnetic Field-Induced Cognitive Benefit? Neuroscience, 185, 135–149. 10.1016/j.neuroscience.2011.04.012 21514369

[180] Arendash GW, Sanchez-Ramos J, Mori T, Mamcarz M, Lin X, Runfeldt M, (2010) Electromagnetic Field Treatment Protects against and Reverses Cognitive Impairment in Alzheimer’s Disease Mice. Journal of Alzheimer’s Disease, 19, 191–210. 10.3233/jad-2010-1228 20061638

[181] Jeong H, Andersson J, Hess A and Jezzard P (2023) Effect of Subject-Specific Head Morphometry on Specific Absorption Rate Estimates in Parallel-Transmit MRI at 7 T. Magnetic Resonance in Medicine, 89, 2376–2390. 10.1002/mrm.29589 36656151 PMC10952207

[182] Banaceur S, Banasr S, Sakly M and Abdelmelek H (2013) Whole Body Exposure to 2.4 GHz WIFI Signals: Effects on Cognitive Impairment in Adult Triple Transgenic Mouse Models of Alzheimer’s Disease (3xTg-AD). Behavioural Brain Research, 240, 197–201. 10.1016/j.bbr.2012.11.021 23195115

[183] Jeong YJ, Son Y, Choi H, Kim N, Lee Y, Ko Y, (2020) Behavioral Changes and Gene Profile Alterations after Chronic 1,950-MHz Radiofrequency Exposure: An Observation in C57BL/6 Mice. Brain and Behavior, 10, e01815. 10.1002/brb3.1815 32856797 PMC7667305

[184] Kumlin T, Iivonen H, Miettinen P, Juvonen A, van Groen T, Puranen L, (2007) Mobile Phone Radiation and the Developing Brain: Behavioral and Morphological Effects in Juvenile Rats. Radiation Research, 168, 471–479. 10.1667/rr1002.1 17903040

[185] Wang K, Lu J, Xing Z, Zhao Q, Hu L, Xue L, (2017) Effect of 1.8 GHz Radiofrequency Electromagnetic Radiation on Novel Object Associative Recognition Memory in Mice. Scientific Reports, 7, Article No. 44521. 10.1038/srep44521 PMC5355939 28303965

[186] Son Y, Kim JS, Jeong YJ, Jeong YK, Kwon JH, Choi H, (2018) Long-Term RF Exposure on Behavior and Cerebral Glucose Metabolism in 5xFAD Mice. Neuroscience Letters, 666, 64–69. 10.1016/j.neulet.2017.12.042 29273398

[187] Hu Y, Lai J, Wan B, Liu X, Zhang Y, Zhang J, (2016) Long-Term Exposure to ELF-MF Ameliorates Cognitive Deficits and Attenuates Tau Hyperphosphorylation in 3xTg AD Mice. NeuroToxicology, 53, 290–300. 10.1016/j.neuro.2016.02.012 26945731

[188] Liu X, Zuo H, Wang D, Peng R, Song T, Wang S, (2015) Improvement of Spatial Memory Disorder and Hippocampal Damage by Exposure to Electromagnetic Fields in an Alzheimer’s Disease Rat Model. PLOS ONE, 10, e0126963. 10.1371/journal.pone.0126963 25978363 PMC4433192

[189] Akbarnejad Z, Esmaeilpour K, Shabani M, Asadi-Shekaari M, Saeedi goraghani M and Ahmadi-Zeidabadi M (2017) Spatial Memory Recovery in Alzheimer’s Rat Model by Electromagnetic Field Exposure. International Journal of Neuroscience, 128, 691–696. 10.1080/00207454.2017.1411353 29185809

[190] de Pomerai D, Daniells C, David H, Allan J, Duce I, Mutwakil M, (2000) Non-Thermal Heat-Shock Response to Microwaves. Nature, 405, 417–418. 10.1038/35013144 10839528

[191] Shallom JM, Di Carlo AL, Ko D, Penafiel LM, Nakai A and Litovitz TA (2002) Microwave Exposure Induces Hsp70 and Confers Protection against Hypoxia in Chick Embryos. Journal of Cellular Biochemistry, 86, 490–496. 10.1002/jcb.10243 12210755

[192] Weisbrot D, Lin H, Ye L, Blank M and Goodman R (2003) Effects of Mobile Phone Radiation on Reproduction and Development in drosophila Melanogaster. Journal of Cellular Biochemistry, 89, 48–55. 10.1002/jcb.10480 12682907

[193] Arendash G, Cao C, Abulaban H, Baranowski R, Wisniewski G, Becerra L, (2019) A Clinical Trial of Transcranial Electromagnetic Treatment in Alzheimer’s Disease: Cognitive Enhancement and Associated Changes in Cerebrospinal Fluid, Blood, and Brain Imaging. Journal of Alzheimer’s Disease, 71, 57–82. 10.3233/jad-190367 PMC6839500 31403948

[194] Arendash G, Abulaban H, Steen S, Andel R, Wang Y, Bai Y, (2022) Transcranial Electromagnetic Treatment Stops Alzheimer’s Disease Cognitive Decline over a 2½-Year Period: A Pilot Study. Medicines, 9, Article No. 42. 10.3390/medicines9080042 PMC9416517 36005647

[195] Cao C, Abulaban H, Baranowski R, Wang Y, Bai Y, Lin X, (2022) Transcranial Electromagnetic Treatment “Rebalances” Blood and Brain Cytokine Levels in Alzheimer’s Patients: A New Mechanism for Reversal of Their Cognitive Impairment. Frontiers in Aging Neuroscience, 14, Article ID: 829049. 10.3389/fnagi.2022.829049 PMC9108275 35585867

[196] Söderqvist F, Hardell L, Carlberg M and Mild KH (2010) Radiofrequency Fields, Transthyretin, and Alzheimer’s Disease. Journal of Alzheimer’s Disease, 20, 599–606. 10.3233/jad-2010-1395 20164553

[197] Sandyk R (1994) Alzheimer’s Disease: Improvement of Visual Memory and Visuoconstructive Performance by Treatment with Picotesla Range Magnetic Fields. International Journal of Neuroscience, 76, 185–225. 10.3109/00207459408986003 7960477

[198] He G-L, Liu Y, Li M, Chen C-H, Gao P, Yu Z-P and Yang X-S (2014) The Amelioration of Phagocytic Ability in Microglial Cells by Curcumin through the Inhibition of EMF-Induced Pro-Inflammatory Responses. Journal of Neuroinflammation, 11, Article No. 49. 10.1186/1742-2094-11-49 PMC3994595 24645646

[199] Barthélémy A, Mouchard A and Villégier A (2016) Glial Markers and Emotional Memory in Rats Following Cerebral Radiofrequency Exposures. 2016 IEEE Radio and Antenna Days of the Indian Ocean (RADIO), Reunion, 10–13 October 2016, 1–2. 10.1109/radio.2016.7772023 27696165

[200] Jiang D, Li J, Zhang J, Xu S, Kuang F, Lang H, (2013) Electromagnetic Pulse Exposure Induces Overexpression of Beta Amyloid Protein in Rats. Archives of Medical Research, 44, 178–184. 10.1016/j.arcmed.2013.03.005 23523687

[201] Akbarnejad Z, Esmaeilpour K, Shabani M, Asadi-Shekaari M, Saeedi Goraghani M and Ahmadi-Zeidabadi M (2018) Spatial Memory Recovery in Alzheimer’s Rat Model by Electromagnetic Field Exposure. International Journal of Neuroscience, 128, 691–696. 10.1080/00207454.2017.1411353 29185809

[202] Qiao S, Peng R, Yan H, Gao Y, Wang C, Wang S, (2014) Reduction of Phosphorylated Synapsin I (ser-553) Leads to Spatial Memory Impairment by Attenuating GABA Release after Microwave Exposure in Wistar Rats. PLOS ONE, 9, e95503. 10.1371/journal.pone.0095503 24743689 PMC3990695

[203] Prochnow N, Gebing T, Ladage K, Krause-Finkeldey D, El Ouardi A, Bitz A, (2011) Electromagnetic Field Effect or Simply Stress? Effects of UMTS Exposure on Hippocampal Longterm Plasticity in the Context of Procedure Related Hormone Release. PLOS ONE, 6, e19437. 10.1371/journal.pone.0019437 21573218 PMC3088670

[204] Wang H, Peng R, Zhou H, Wang S, Gao Y, Wang L, (2013) Impairment of Long-Term Potentiation Induction Is Essential for the Disruption of Spatial Memory after Microwave Exposure. International Journal of Radiation Biology, 89, 1100–1107. 10.3109/09553002.2013.817701 23786183

[205] Wang H, Peng R, Zhao L, Wang S, Gao Y, Wang L, (2015) The Relationship between NMDA Receptors and Microwave-Induced Learning and Memory Impairment: A Long-Term Observation on Wistar Rats. International Journal of Radiation Biology, 91, 262–269. 10.3109/09553002.2014.988893 25426698

[206] Wang H, Tan S, Xu X, Zhao L, Zhang J, Yao B, (2017) Long Term Impairment of Cognitive Functions and Alterations of NMDAR Subunits after Continuous Microwave Exposure. Physiology & Behavior, 181, 1–9. 10.1016/j.physbeh.2017.08.022 28866028

[207] Foroozandeh E, Naeini MS, Ahadi H and Foroozandeh J (2011) Effects of 90min Exposure to 8mT Electromagnetic Fields on Memory in Mice. Journal of American Science, 7, 58–61.

[208] Yang X, He G, Hao Y, Chen C, Li M, Wang Y, (2010) The Role of the JAK2-STAT3 Pathway in Pro-Inflammatory Responses of EMF-Stimulated N9 Microglial Cells. Journal of Neuroinflammation, 7, Article No. 54. 10.1186/1742-2094-7-54 PMC2945324 20828402

[209] Cleary SF, Cao G, Liu L, Egle PM and Shelton KR (1997) Stress Proteins Are Not Induced in Mammalian Cells Exposed to Radiofrequency or Microwave Radiation. Bioelectromagnetics, 18, 499–505. 10.1002/(sici)1521-186x(1997)18:7<499::aid-bem5>3.0.co;2-y 9338631

[210] Regel SJ, Tinguely G, Schuderer J, Adam M, Kuster N, Landolt H, (2007) Pulsed Radio-Frequency Electromagnetic Fields: Dose-Dependent Effects on Sleep, the Sleep EEG and Cognitive Performance. Journal of Sleep Research, 16, 253–258. 10.1111/j.1365-2869.2007.00603.x 17716273

[211] Regel SJ, Gottselig JM, Schuderer J, Tinguely G, Rétey JV, Kuster N, (2007) Pulsed Radio Frequency Radiation Affects Cognitive Performance and the Waking Electroencephalogram. NeuroReport, 18, 803–807. 10.1097/wnr.0b013e3280d9435e 17471070

[212] Kim JH, Yu D, Huh YH, Lee EH, Kim H and Kim HR (2017) Long-Term Exposure to 835 MHz RF-EMF Induces Hyperactivity, Autophagy and Demyelination in the Cortical Neurons of Mice. Scientific Reports, 7, Article No. 41129. 10.1038/srep41129 PMC5247706 28106136

[213] I.S. C95.1 (2019) Safety Levels with Respect to Human Exposure to Electric, Magnetic, and Electromagnetic Fields, 0 Hz to 300 GHz.

[214] Lanni I, Chiacchierini G, Papagno C, Santangelo V and Campolongo P (2024) Treating Alzheimer’s Disease with Brain Stimulation: From Preclinical Models to Non-Invasive Stimulation in Humans. Neuroscience & Biobehavioral Reviews, 165, Article ID: 105831. 10.1016/j.neubiorev.2024.105831 39074672

[215] Ribeiro FM, Camargos E.R.d.S., Souza L.C.d. and Teixeira AL (2013) Animal Models of Neurodegenerative Diseases. Revista Brasileira de Psiquiatria, 35, S82–S91. 10.1590/1516-4446-2013-1157 24271230

[216] Guerriero F, Botarelli E, Mele G, Polo L, Zoncu D, Renati P, (2015) An Innovative Intervention for the Treatment of Cognitive Impairment-Emisymmetric Bilateral Stimulation Improves Cognitive Functions in Alzheimer’s Disease and Mild Cognitive Impairment: An Open-Label Study. Neuropsychiatric Disease and Treatment, 11, 2391–2404. 10.2147/ndt.s90966 26425094 PMC4581783

[217] Son Y, Park H, Jeong YJ, Choi H, Kim N and Lee H (2023) Long-Term Radiofrequency Electromagnetic Fields Exposure Attenuates Cognitive Dysfunction in 5×FAD Mice by Regulating Microglial Function. Neural Regeneration Research, 18, 2497–2503. 10.4103/1673-5374.371379 37282482 PMC10360091

[218] Zhi W, Zou Y, Ma L, He S, Guo Z, Zhao X, (2023) 900 MHz Electromagnetic Field Exposure Relieved AD-Like Symptoms on APP/PS1 Mice: A Potential Non-Invasive Strategy for AD Treatment. Biochemical and Biophysical Research Communications, 658, 97–106. 10.1016/j.bbrc.2023.03.083 37030070

[219] Komaki A, Salehi I, Keymoradzadeh A, Taheri Azandaryani M and Golipoor Z (2021) Effect of Long-Term Exposure to Extremely Low-Frequency Electromagnetic Fields on *β*-Amyloid Deposition and Microglia Cells in an Alzheimer Model in Rats. Journal of Guilan University of Medical Sciences, 30, 218–229. 10.32598/jgums.30.3.1609.2

[220] Zhang S, (2024) Effects of 2.4 GHz Radiofrequency Electromagnetic Field Exposure on Hippocampal Proteins in APP/PS1 Mice.

[221] Teranishi M, Ito M, Huang Z, Nishiyama Y, Masuda A, Mino H, (2024) Extremely Low-Frequency Electromagnetic Field (ELF-EMF) Increases Mitochondrial Electron Transport Chain Activities and Ameliorates Depressive Behaviors in Mice. International Journal of Molecular Sciences, 25, Article No. 11315. 10.3390/ijms252011315 PMC11508854 39457098

[222] Kim JH, Yu D, Kim H, Huh YH, Cho S, Lee J, (2017) Exposure to 835 MHz Radiofrequency Electromagnetic Field Induces Autophagy in Hippocampus but Not in Brain Stem of Mice. Toxicology and Industrial Health, 34, 23–35. 10.1177/0748233717740066 29166827

[223] Guo R-W, (2024) Rotating Magnetic Field Inhibits A*β* Protein Aggregation and Alleviates Cognitive Impairment in Alzheimer’s Disease Mice. Zoological Research, 45, 924. 39021081 10.24272/j.issn.2095-8137.2024.034 PMC11298676

[224] Zhang J, Chen Y, Zhao Y, Wang P, Ding H, Liu C, (2023) Terahertz Irradiation Improves Cognitive Impairments and Attenuates Alzheimer’s Neuropathology in the APPSWE/PS1DE9 Mouse: A Novel Therapeutic Intervention for Alzheimer’s Disease. Neuroscience Bulletin, 40, 857–871. 10.1007/s12264-023-01145-3 37971654 PMC11250709

[225] Moya-Gómez A, Font LP, Burlacu A, Alpizar YA, Cardonne MM, Brône B, (2023) Extremely Low-Frequency Electromagnetic Stimulation (ELF-EMS) Improves Neurological Outcome and Reduces Microglial Reactivity in a Rodent Model of Global Transient Stroke. International Journal of Molecular Sciences, 24, Article No. 11117. 10.3390/ijms241311117 PMC10342400 37446295

[226] Abkhezr H, Mohaddes G, Nikniaz Z, Abbasalizad Farhangi M, Heydari H and Nikniaz L (2023) The Effect of Extremely Low Frequency Electromagnetic Field on Spatial Memory of Mice and Rats: A Systematic Review. Learning and Motivation, 81, Article ID: 101873. 10.1016/j.lmot.2023.101873

[227] Eskandani R and Zibaii MI (2023) Unveiling the Biological Effects of Radio-Frequency and Extremely-Low Frequency Electromagnetic Fields on the Central Nervous System Performance. BioImpacts, 14, Article No. 30064. 10.34172/bi.2023.30064 PMC11298025 39104617

[228] Perez FP, Morisaki J, Kanakri H and Rizkalla M (2024) Electromagnetic Field Stimulation Therapy for Alzheimer’s Disease. Neurology (Chic), 3, 1020. 38699565 PMC11064876

[229] Jiang D, Li J, Zhang J, Xu S, Kuang F, Lang H, (2016) Long-Term Electromagnetic Pulse Exposure Induces Abeta Deposition and Cognitive Dysfunction through Oxidative Stress and Overexpression of APP and BACE1. Brain Research, 1642, 10–19. 10.1016/j.brainres.2016.02.053 26972535

[230] Zhang Y, Liu X, Zhang J and Li N (2014) Short-Term Effects of Extremely Low Frequency Electromagnetic Fields Exposure on Alzheimer’s Disease in Rats. International Journal of Radiation Biology, 91, 28–34. 10.3109/09553002.2014.954058 25118893

[231] Bouji M, Lecomte A, Gamez C, Blazy K and Villégier A (2019) Impact of Cerebral Radiofrequency Exposures on Oxidative Stress and Corticosterone in a Rat Model of Alzheimer’s Disease. Journal of Alzheimer’s Disease, 73, 467–476. 10.3233/jad-190593 31796670

[232] Son Y, Jeong YJ, Kwon JH, Choi H, Pack J, Kim N, (2016) 1950 MHz Radiofrequency Electromagnetic Fields Do Not Aggravate Memory Deficits in 5xFAD Mice. Bioelectromagnetics, 37, 391–399. 10.1002/bem.21992 27434853 PMC5108492

[233] Perez FP, Walker B, Morisaki J, Kanakri H and Rizkalla M (2025) Neurostimulation Devices to Treat Alzheimer’s Disease. Exploration of Neuroscience, 4, Article ID: 100674. 10.37349/en.2025.100674 PMC11904933 40084342

[234] Messori C, Prinzera SV and Di Bardone FB (2019) The Super-Coherent State of Biological Water. Open Access Library Journal, 6, 1–5. 10.4236/oalib.1105236

[235] Madl P and Renati P (2023) Quantum Electrodynamics Coherence and Hormesis: Foundations of Quantum Biology. International Journal of Molecular Sciences, 24, Article No. 14003. 10.3390/ijms241814003 PMC10530466 37762305

[236] Huang Z, Ito M, Zhang S, Toda T, Takeda J, Ogi T, (2023) Extremely Low-Frequency Electromagnetic Field Induces Acetylation of Heat Shock Proteins and Enhances Protein Folding. Ecotoxicology and Environmental Safety, 264, Article ID: 115482. 10.1016/j.ecoenv.2023.115482 37717354

[237] Morotomi-Yano K, Oyadomari S, Akiyama H and Yano K (2012) Nanosecond Pulsed Electric Fields Act as a Novel Cellular Stress That Induces Translational Suppression Accompanied by eIF2*α* Phosphorylation and 4E-BP1 Dephosphorylation. Experimental Cell Research, 318, 1733–1744. 10.1016/j.yexcr.2012.04.016 22652449

[238] Kim K, Lee YS, Kim N, Choi H and Lim K (2022) 5G Electromagnetic Radiation Attenuates Skin Melanogenesis *in Vitro* by Suppressing ROS Generation. Antioxidants, 11, Article No. 1449. 10.3390/antiox11081449 PMC9331092 35892650

[239] Lang BJ, Guerrero ME, Prince TL, Okusha Y, Bonorino C and Calderwood SK (2021) The Functions and Regulation of Heat Shock Proteins; Key Orchestrators of Proteostasis and the Heat Shock Response. Archives of Toxicology, 95, 1943–1970. 10.1007/s00204-021-03070-8 34003342

[240] Hu C, Yang J, Qi Z, Wu H, Wang B, Zou F, (2022) Heat Shock Proteins: Biological Functions, Pathological Roles, and Therapeutic Opportunities. MedComm, 3, e161. 10.1002/mco2.161 35928554 PMC9345296

[241] Usselman RJ, Hill I, Singel DJ and Martino CF (2014) Spin Biochemistry Modulates Reactive Oxygen Species (ROS) Production by Radio Frequency Magnetic Fields. PLOS ONE, 9, e93065. 10.1371/journal.pone.0093065 24681944 PMC3969378

[242] Zeng Y, Shen Y, Hong L, Chen Y, Shi X, Zeng Q, (2017) Effects of Single and Repeated Exposure to a 50-Hz 2-Mt Electromagnetic Field on Primary Cultured Hippocampal Neurons. Neuroscience Bulletin, 33, 299–306. 10.1007/s12264-017-0113-6 28265899 PMC5567517

[243] Benassi B, Filomeni G, Montagna C, Merla C, Lopresto V, Pinto R, (2015) Extremely Low Frequency Magnetic Field (ELF-MF) Exposure Sensitizes SH-SY5Y Cells to the Pro-Parkinson’s Disease Toxin MPP+. Molecular Neurobiology, 53, 4247–4260. 10.1007/s12035-015-9354-4 26223801

[244] Schuermann D and Mevissen M (2021) Manmade Electromagnetic Fields and Oxidative Stress—Biological Effects and Consequences for Health. International Journal of Molecular Sciences, 22, Article No. 3772. 10.3390/ijms22073772 PMC8038719 33917298

[245] Kang KA, Lee HC, (2013) Effects of Combined Radiofrequency Radiation Exposure on Levels of Reactive Oxygen Species in Neuronal Cells. Journal of Radiation Research, 55, 265–276. 10.1093/jrr/rrt116 24105709 PMC3951078

[246] de Gannes FP, Haro E, Hurtier A, Taxile M, Ruffié G, Billaudel B, (2011) Effect of Exposure to the Edge Signal on Oxidative Stress in Brain Cell Models. Radiation Research, 175, 225–230. 10.1667/rr2320.1 21268716

[247] Alejandro M, Herlinda B, Aparicio-Bautista DI, Siddhartha M, Overduin M and Basurto-Islas G (2025) Molecular Mechanisms Associated with the Interaction of External Electromagnetic Fields in Protein Dynamics and Aggregation: A Focus on Amyloid-*β* Peptide. Progress in Biomedical Engineering, 7, Article ID: 032010. 10.1088/2516-1091/adea02 40588003

[248] Saikia J, Pandey G, Sasidharan S, Antony F, Nemade HB, Kumar S, (2019) Electric Field Disruption of Amyloid Aggregation: Potential Noninvasive Therapy for Alzheimer’s Disease. ACS Chemical Neuroscience, 10, 2250–2262. 10.1021/acschemneuro.8b00490 30707008

[249] Bhattacharjee S, Choi J, Park S, Shim K, Baek C, Han H, (2025) *In Vitro* Treatment of Alzheimer’s Disease by Disintegrating Amyloid-*β* Using Electromagnetic Waves. IEEE Transactions on Antennas and Propagation, 73, 6788–6799. 10.1109/tap.2025.3568638

[250] Muscat S, Stojceski F and Danani A (2020) Elucidating the Effect of Static Electric Field on Amyloid Beta 1–42 Supramolecular Assembly. Journal of Molecular Graphics and Modelling, 96, Article ID: 107535. 10.1016/j.jmgm.2020.107535 31978828

[251] Vargas-Rosales PA, D’Addio A, Zhang Y and Caflisch A (2023) Disrupting Dimeric *β*-Amyloid by Electric Fields. ACS Physical Chemistry Au, 3, 456–466. 10.1021/acsphyschemau.3c00021 37780539 PMC10540290

[252] Salehi N, Lohrasebi A and Bordbar AK (2023) Preventing the Amyloid-Beta Peptides Accumulation on the Cell Membrane by Applying GHz Electric Fields: A Molecular Dynamic Simulation. Journal of Molecular Graphics and Modelling, 123, Article ID: 108516. 10.1016/j.jmgm.2023.108516 37216829

[253] Allen RM, Scanlan JM and Gama-Chonlon L (2023) Bilateral Rtms Shows No Advantage in Depression nor in Comorbid Depression and Anxiety: A Naturalistic Study. Psychiatric Quarterly, 95, 107–120. 10.1007/s11126-023-10062-7 38127248

[254] Yang Y, Hsieh S, Chang H, Sung J, Chuu C, Yen C, (2022) Gamma Frequency Inhibits the Secretion and Aggregation of Amyloid-*β* and Decreases the Phosphorylation of mTOR and Tau Proteins *in Vitro*. Journal of Alzheimer’s Disease, 90, 917–928. 10.3233/jad-220307 36189589

[255] Lobyntseva A, Ganaiem M, Ivashko-Pachima Y, Barnstable CJ, Weisinger B, Parabucki A, (2025) Extremely Low-Frequency and Low-Intensity Electromagnetic Field Technology (ELF-EMF) Sculpts Microtubules. European Journal of Neuroscience, 61, e70023. 10.1111/ejn.70023 39966100 PMC11835790

[256] Todorova N, Bentvelzen A and Yarovsky I (2020) Electromagnetic Field Modulates Aggregation Propensity of Amyloid Peptides. The Journal of Chemical Physics, 152, Article ID: 035104. 10.1063/1.5126367 31968963

[257] Toschi F, Lugli F, Biscarini F and Zerbetto F (2008) Effects of Electric Field Stress on a *β*-Amyloid Peptide. The Journal of Physical Chemistry B, 113, 369–376. 10.1021/jp807896g 19063666

[258] Lin JC (2012) Electromagnetic Fields in Biological Systems. Taylor & Francis.

[259] Lambert N, Chen Y, Cheng Y, Li C, Chen G and Nori F (2012) Quantum Biology. Nature Physics, 9, 10–18. 10.1038/nphys2474

[260] Marino AA and Becker RO (1977) Biological Effects of Extremely Low Frequency Electric and Magnetic Fields: A Review. Physiological Chemistry and Physics, 9, 131–147. 414240

[261] Semchenko IV, Mikhalka IS, Khakhomov SA, Samofalov AL and Balmakou AP (2022) DNA-Like Helices as Nanosized Polarizers of Electromagnetic Waves. Frontiers in Nanotechnology, 4, Article ID: 794213. 10.3389/fnano.2022.794213

[262] Li M, Liu M and Sha Y (2021) Induced and Inversed Circularly Polarized Luminescence of Achiral Thioflavin T Assembled on Peptide Fibril. Small, 18, Article ID: 2106130. 10.1002/smll.202106130 34881501

[263] Zhang Z, Fan F, Shi W, Zhang T and Chang S (2021) Terahertz Circular Polarization Sensing for Protein Denaturation Based on a Twisted Dual-Layer Metasurface. Biomedical Optics Express, 13, 209–221. 10.1364/boe.443473 35154865 PMC8803037

[264] Perez F, Morisaki J, Kanakri H, Rizkalla M and Abdalla A (2025) A Novel Design of a Portable Birdcage via Meander Line Antenna (MLA) to Lower Beta Amyloid (a*β*) in Alzheimer’s Disease. IEEE Journal of Translational Engineering in Health and Medicine, 13, 158–173. 10.1109/jtehm.2025.3559693 40657533 PMC12251030

[265] Wyszkowska J, Jankowska M and Gas P (2019) Electromagnetic Fields and Neurodegenerative Diseases. Przegląd Elektrotechniczny, 95, 131–135. 10.15199/48.2019.01.33

[266] Fymat AL (2020) Electromagnetic Therapy for Neurological and Neurodegenerative Diseases: II. Deep Brain Stimulation. Open Access Journal of Neurology & Neurosurgery, 13, Article No. 555855. 10.19080/oajnn.2020.13.555855

